# Whole-genome comparative analysis of *Campylobacter jejuni* strains isolated from patients with diarrhea in northeastern Poland

**DOI:** 10.1186/s13099-019-0313-x

**Published:** 2019-06-19

**Authors:** Krzysztof Fiedoruk, Tamara Daniluk, Dorota Rozkiewicz, Elzbieta Oldak, Suhanya Prasad, Izabela Swiecicka

**Affiliations:** 1 Department of Microbiology, Medical University of Bialystok, Bialystok, Poland; 2 Department of Pediatric Infectious Diseases, Medical University of Bialystok, University Children’s Hospital, Bialystok, Poland; 3 Department of Microbiological and Nanobiomedical Engineering, Medical University of Bialystok, Bialystok, Poland; 4 Department of Microbiology, University of Bialystok, Bialystok, Poland; 5 Laboratory of Applied Microbiology, University of Bialystok, Bialystok, Poland

**Keywords:** Campylobacteriosis, WGS, MLST, Virulence, Antibiotic resistance

## Abstract

**Background:**

*Campylobacter jejuni* is the leading cause of bacterial gastroenteritis (campylobacteriosis) in humans worldwide, and the most frequent pathogen associated with Guillain-Barré syndrome (GBS) and Miller-Fisher syndrome (MFS). The study was designed in order to assess similarities between genomes of *Campylobacter jejuni* strains, isolated from children suffering from acute diarrhea in northeastern Poland, in comparison to *C.* *jejuni* genomes stored in public databases. The analysis involved phylogeny, resistome and virulome. In addition, the *Campylobacter* PubMLST database was used to estimate the prevalence of the analyzed *C. jejuni* sequence type (STs) in other countries.

**Results:**

*Campylobacter jejuni* ST50, ST257 and ST51 represented 5.3%, 4.5% and 2.2% of the PubMLST records, respectively. Overall, strains representing the STs showed common resistance to tetracyclines (51.3%) and fluoroquinolones (31.8%), mediated through the *tetO* gene (98.2%) and point mutation (T86I) in the *gyrA* gene (100%). However, the latter was present in all our isolates. The major differences in virulence patterns concerned serotypes, lipooligosaccharide (LOS) classes and certain clinically relevant genes.

**Conclusions:**

*Campylobacter jejuni* ST50, ST51 and ST257 are among the top ten of STs isolated in Europe. WGS revealed diversity of serotypes and LOS classes in ST50 strains, that deserves further clinical and epidemiological investigations as it might be related to a risk of post-infectious neurological sequels such as Guillain-Barré syndrome. Additionally, the results implicate lower pathogenic potential and distinct transmission chains or reservoirs for *C. jejuni* ST51 isolates responsible for campylobacteriosis in northeastern Poland.

**Electronic supplementary material:**

The online version of this article (10.1186/s13099-019-0313-x) contains supplementary material, which is available to authorized users.

## Background

*Campylobacter jejuni* is the leading cause of bacterial gastroenteritis (campylobacteriosis) in humans worldwide, with more than 200,000 annual cases in European Union (EU) [1, 2]. The natural reservoirs of *C. jejuni* are intestinal tracts of many wild and agriculture-associated birds and mammals. Therefore, faecal contamination from carrier animals is considered to be a primary source of this pathogen in the environment and food products. Although the transmission routes of *C. jejuni* are not fully recognized, chickens are considered to be the major source for transmission to humans. In addition, outbreaks of campylobacteriosis are often linked to consumption of unpasteurized milk and contaminated water [3].

In general, campylobacteriosis is a self-limiting illness, however, rare but life-threatening neurological sequels such as Guillain-Barré syndrome (GBS) and Miller-Fisher syndrome (MFS) may occur in patients [4]. For instance, it was estimated that 31.0% of the GBS cases may be ascribed to a previous campylobacteriosis [5]*.* Yet in contrast to other bacterial enteropathogens, *C. jejuni* does not possess numerous classical virulence factors. Cytolethal distending enterotoxin (Cdt) is the only virulence determinant located on *C. jejuni* chromosome, however, its role in the pathogenesis is still not clear [6]. Nevertheless (i) flagella based motility and chemotaxis as well as secretion of invasive antigens (Cia) (ii) polysaccharide capsule (CPS) (iii) lipooligosaccharide (LOS), and (iv) various proteins associated with adhesion, colonization and infection of host cells, were recognized as important factors for *C. jejuni* pathogenicity [7, 8]. Additionally, CPS and LOS are also implicated with post-gastroenteritis GBS and MFS [4, 9]. Therefore, the identification and profiling of *C. jejuni* virulence determinants are crucial for risk assessment of infections caused by this pathogen.

Although antimicrobial therapy is not routinely recommended to treat campylobacteriosis, in severe and prolonged or immunocompromised cases, fluoroquinolones and macrolides are agents of choice [10]. Also, tetracyclines and aminoglycosides can be used as alternatives. However, an increasing resistance of *C. jejuni*, in particular to fluoroquinolones, in recent years is alarming. As a result, in 2017 the World Health Organization listed fluoroquinolone-resistant *Campylobacter* spp. as one of the six high priority pathogens for research and development of new antibiotics [11].

Multilocus sequence typing (MLST), based on sequence comparison of seven housekeeping genes defined as sequence types (STs) and clonal complexes (CCs), has been an essential tool in studying of *C. jejuni* phylogeny and epidemiology [12]. However, MLST does not include medically relevant information such as the virulence or antibiotic resistance determinants, also known as virulome and resistome [12, 13]. In addition, since *C. jejuni* is genetically variable pathogen with high level of horizontal gene exchange and recombination, even strains representing the same STs may possess distinct virulence patterns [9, 14]. At present, whole-genome sequencing (WGS) is considered as the most informative and discriminative typing method of bacterial pathogens [14–16], allowing for comprehensive phylogenetic analyses of numerous traits associated with virulence [17, 18] or antibiotic resistance [16, 19].

In this study we applied WGS in order (i) to characterize *C. jejuni* strains isolated from children with acute diarrhea in northeastern Poland, and (ii) to compare their virulence and antibiotic resistance patterns with phylogenetically related, i.e. representing the same STs, *C. jejuni* strains from other parts of the world.

## Results

### Sequence types (STs), serotypes, phylogenetic relatedness and pan-genome

Four *C. jejuni* isolates from children with diarrhea were classified into three STs, ST50 (strains KF017 and KF042), ST51 (strain KF070), and ST257 (strain KF045). According to the *Campylobacter* PubMLST database involving 6977 distinct *C. jejuni* STs, ST50, ST257, and ST51 were among the top ten STs (Table 1). Likewise, among 139 various STs from Poland (289 records), ST257 (8.6%) and ST50 (3.5%) occupied the second and third place respectively, outdistanced by ST6411 (10.0%). Also, in the PATRIC genome database ST50, ST257 and ST51 represented twelve the most commonly sequenced *C. jejuni* STs (Additional file 1: Table S3).

**Table 1:** The ten most common *C. jejuni* STs collected in the *Campylobacter* PubMLST database comprising 58,179 records

ST	Number of strains	% of the total STs^a^	Clonal Complex
21	3517	6.0	CC-21
45	3141	5.4	CC-45
50	3064	5.3	CC-21
257	2623	4.5	CC-257
48	2478	4.3	CC-48
53	1483	2.5	CC-21
61	1296	2.2	CC-61
19	1270	2.2	CC-21
51	1270	2.2	CC-443
354	1171	2.0	CC-354
42	1053	1.8	CC-42

^a^6977 is the total number of STs, profiles without ascribed ST (n = 53) were excluded from the comparison

**Fig. 1:**
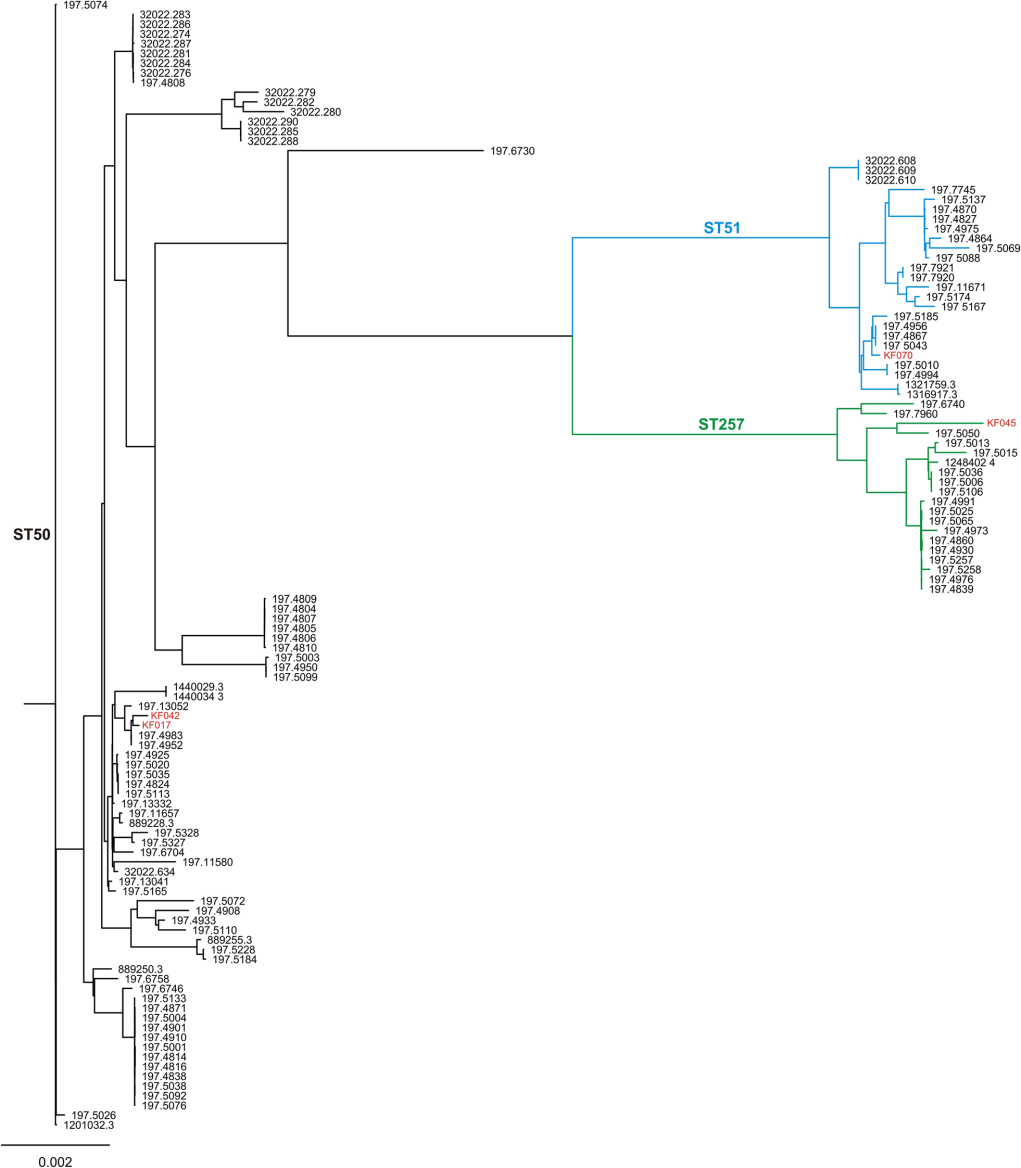
Phylogenetic tree of *C. jejuni* ST50, ST51 and ST257 constructed based on core genes comparison using Roary and RAxML software. Black, blue and green colors of the tree branches indicate ST50, ST51 and ST257 strains, respectively. The isolates from northeastern Poland are in red

All strains classified as ST51 and ST257 belonged to the HS37 and HS11 serotypes, respectively. On the contrary, ST50 strains were represented by five serotypes, where HS8c predominated (49.3%), including our two isolates KF042 and KF017, followed by HS1 (33.8%), HS2 (8.4%), HS10 (4.2%), and HS5c (4.2%) (Additional file 1: Table S1). This diversity was reflected by the phylogenetic analysis that revealed several discrete clusters formed by the ST50 strains (Fig. 1).

The pan-genome analysis revealed a total of 5961 genes, including 907 and 376 of the core and the shell core genes, respectively (Additional file 1: Fig. S1). Additional data regarding the pan-genome analysis, i.e. frequency of genes and their presence or absence in genomes, were included in Additional file 1: Fig. S2 and S3.

**Fig. 2:**
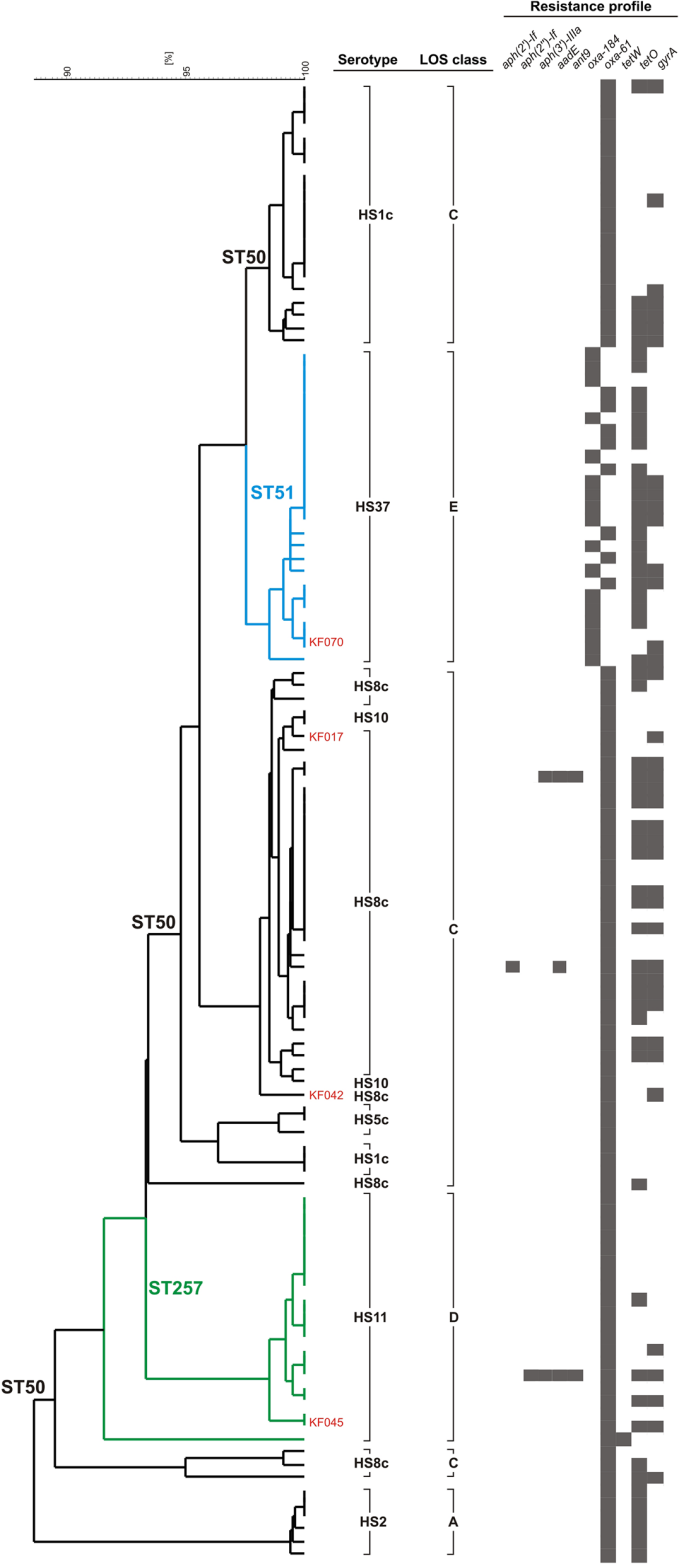
Dendrogram representing similarity of virulence patterns of *C. jejuni* ST50, ST51 and ST257 strains. The dendrogram was constructed based on presence/absence of virulence genes (n = 143) using UPGMA method and Dice coefficient in NTSYS-pc software. Black, blue and green colors of the tree branches indicate ST50, ST51 and ST257 strains, respectively. The isolates from northeastern Poland are shown in red

## Resistome

Overall, based on in silico analysis in *C. jejuni* isolates from northeastern Poland we identified resistance determinants to three groups of antibiotics, beta-lactams (*bla*_*oxa-61*_ or *bla*_*oxa-184*_), tetracyclines (*tetO*), and fluoroquinolones (point mutation T86I in *gyrA*) (Table 2, Fig. 2), which was consistent with their in vitro antibiotic resistance patterns. The same resistance markers were noted in the remaining *C. jejuni* genomes, which only sporadically possessed aminoglycoside resistance genes (three strains). In addition, in one genome (*C. jejuni* BIGS0022) *tetW* was present instead of *tetO*. No mutations in 23S rRNA or other genes (*cmeR*, *rplV*, *rpsL*) associated with macrolide or spectinomycin resistance were detected. Therefore, altogether twelve antibiotic resistance patterns were distinguished (Table 2).

In total, tetracycline and fluoroquinolone resistance determinants were present in 51.3% and 31.8% of the genomes, respectively. Conversely, *tetO* was detected only in one of our strains (KF045), but all have T86I mutation in *gyrA*. Furthermore, in 58.6% of *C. jejuni* strains *tetO* has plasmid origin, since it is located on contigs sharing similarity with pTet plasmid of *C. jejuni* 81–176 or similar plasmids (Additional file 1: Fig. S4). In addition, 14 variants of TetO protein were noted with amino acid sequence identity varied from 93 to 99% (Additional file 1: Fig. S5). Interestingly, in *C. jejuni* KF045 *tetO* is localized in unique chromosomal position in comparison to other strains representing ST257, ST50, and ST51 (Additional file 1: Fig. S6).

As aforementioned, aminoglycoside resistance genes were noted only in three genomes, two representing ST50 (*aadE*, *ant9*, and *aph(3′)-IIIa* or *aadE* and *aph(2′)-If*) and one ST257 (*aadE*, *ant9*, *aph(3′)-IIIa* and *aph(2′')-If*) (Table 2). In all cases the genes seem to be located on plasmids, e.g. similar to plasmid pGMI16-00 from *C. jejuni* strain CFSAN054107 (Additional file 1: Fig. S7).

Although the resistance patterns were not connected with a particular ST, *tetO* was more common in genomes belonging to ST51 (84.0%) than in ST50 (47.8%) or ST257 (20%). Furthermore, 32.0% of ST51 genomes possess *bla*_*oxa-61*_, like all ST50 and ST257, instead of *bla*_*oxa-184*_ (Table 2).

**Table 2:** Antibiotic resistance determinants and profiles in genomes (n = 116) of *C. jejuni* ST50, ST51 and ST257

Profile	Resistance determinants										ST number		
	*bla*_*oxa-61*_	*bla*_*oxa-184*_	*tetO*	*tet*W	*gyrA* ^*(T86I)*^	*aph(2′)-If*	*aph(2′')-If*	*aph(3′)-IIIa*	*ant*9	*aad*E	ST50	ST51	ST257
1	+										34		14
2	+		+		+						21	1	2^a^
3	+		+								10	7	1
4		+	+									7	
5		+	+		+							6	
6	+				+						4^a^		1
7		+										3	
8	+		+		+	+				+	1		
9	+		+		+		+	+	+	+			1
10	+		+		+			+	+	+	1		
11	+			+									1
12		+			+							1^a^	

^a^Profiles present in *C. jejuni* strains from northeastern Poland

## Virulome

In general, a majority of the virulence genes, with rare exceptions such as *flaA*, *flaB* or *flaE*, were evenly distributed throughout the genomes of all STs under study (Additional file 1: Table S4). However, the greatest number of virulence genes was noted in strains representing ST50 (mean = 95.1, range = 88–115), followed by ST257 (mean = 90.9, range = 80–93;) and ST51 (mean = 88.4, range = 86–90) strains. The differences were statistically significant between ST50 and ST51 (p = 0.000147). Similarly, the prevalence of the 25 genes recognized as markers of human pathogenic *C. jejuni* strains [17] was significantly higher (p = 0.000114) in the ST50 strains (mean = 23.2, range = 20–24) than ST257 (mean and range = 17) and ST51 (mean = 13.5, range = 11–17) (Additional file 1: Table S5). The differences in virulence patterns (Fig. 2) resulted mainly from a variation in the polysaccharide capsule and LOS biosynthesis loci. The former is also responsible for serotype specificity, and the latter is used to classify *C. jejuni* strains into so-called LOS classes. Hence, all ST51 and ST257 were classified into class E or class D, respectively. Whereas class C was recognized in 91.3% of the ST50 strains, including KF017 and KF042, and class A-specific genes in the remaining genomes. Noteworthy, the latter are characterized also by distinct HS2 serotype (Fig. 2). Finally, plasmids carrying T4SS or T6SS secretion systems were detected only in three genomes, all belonging to ST50.

## Discussion

*Campylobacter jejuni* is a highly diverse pathogen represented currently by around 7000 distinct STs, which are distributed over 44 clonal complexes (CCs) and 2606 singletons (STs without assignment to any CCs). The relative frequency of particular *C. jejuni* genotypes and their diversity may vary between countries, and possibly is influenced by multiple factors, including food sources, animal reservoirs, seasons, levels of zoonotic transmissions, as well as the rate of horizontal gene transfer [7, 20]. Nevertheless, the three STs found among *C. jejuni* Polish isolates, ST50 (CC-21), ST257 (CC-257), and ST51 (CC-443), were in the top ten of *C. jejuni* STs noted in Europe (data from 46,237 records gathered in 28 countries). In fact, ST50 belongs to the largest CC-21, which clusters 23.1% of all STs. Similarly, in Poland the prevalence of ST257 and ST50 is outdistanced by ST6411 only [21]. However, the data from Poland might be skewed by the fact that all strains classified into ST50, ST51 and ST257 were isolated from animals, mostly chickens (86.1%). On the other hand, an isolation of the same STs from animal sources and humans, likely indicates their zoonotic transmission frequently reported by other studies [18, 22]. Actually, CC-257 (ST257) represents so-called ‘specialists’ *C. jejuni* lineage, i.e. strongly associated with certain hosts, chickens in this case for instance [7, 23]. Whereas, CC-21 (ST50) and CC-443 (ST51) are frequently isolated from various animal species, hence considered as ‘generalists’.

Nevertheless, *C. jejuni* pathogenic characteristics, with rare exceptions [24], generally is not attributed to particular phylogenetic lineages [7]. However, we noted that ST50 genomes, followed by ST257, possessed overall the highest number of virulence genes, including those ones considered as typical for human pathogenic *C. jejuni* isolates [17]. Interestingly, Harvala et al. [25] showed that patients infected with *C. jejuni* ST-50 or ST-257 strains were slightly more likely to be hospitalized than those infected with other STs.

Additionally, genetic boundaries between *C. jejuni* genotypes might be readily blurred by horizontal gene transfer and recombination, which are the major driving forces of variability in this species [26, 27]. Indeed, we found a notable variation of ST50 strains in regard to serotype (HS1c, HS2, HS5c, HS8c and HS10) and the LOS class (A and C), the traits mediated via gene diversity in capsular and lipooligosaccharide biosynthesis loci, respectively. Similar observations were made by Skarp et al. [28], who noted microevolution in ST50 strains resulting in their diversification into clusters distinguished by differences in the capsule loci and the distribution of accessory genetic content. Furthermore, based on the core genome analysis the authors revealed tendency for *C. jejuni* ST-50 strains to partially cluster according to their isolation site, i.e. blood vs faeces [28].

Noteworthy, certain serotypes and LOS classes are considered as important risk factors in development of post-infectious neurological sequels such as GBS and MFS [4, 9]. Briefly, the molecular mimicry between sialylated LOS components and gangliosides present on human peripheral nerves explains this relationship. However, genes involved in the synthesis of sialylated LOS are present only in a few (A, B, C, M, and R) among numerous LOS classes (A to S) [29]. Generally, only *C. jejuni* strains with LOS classes A, B and C are frequently isolated from stools of patients with GBS, and the LOS classes A and B are associated with GBS and MFS, respectively [4]. However, this connection is not universal and recently Heikema et al. [9] have recognized the serotypes HS1/44c, HS2, HS4c, HS19, HS23/36c and HS41 as an additional risk factors in GBS. Although we have no information with regard to a potential link between our *C. jejuni* isolates and GBS/MFS, the two ST50 isolates (KF017 and KF042), which partially correspond to this characteristics, i.e. LOS class C but serotype HS8c, seem to pose some risk in GBS/MFS. In contrast, six other ST50 genomes perfectly fit to this LOS/serotype-related GBS/MFS characteristics, i.e. LOS class A and serotype HS2 (Fig. 2, Additional file 1: Table S1). Therefore, MLST-based epidemiology of *C. jejuni* might be insufficient to recognize or to assess all risks associated with *Campylobacter*-related diseases, e.g. GBS or MFS, albeit a connection between these neurologic conditions and infections caused by specific *C. jejuni* strains definitely requires more studies. Despite, *C. jejuni* is a very common enteropathogen in children, it seems that Polish epidemiological data is underestimated and this pathogen is not routinely detected by laboratories in Poland [30], since each year 2–4 GBS cases are noted in children in Podlasie Province and their cause is unknown (personal communication; Department of Neurology, University Children’s Hospital in Bialystok). In our opinion this fact deserves to be publicized and should impact further investigation.

Another remarkable observation is the presence of vitamin B5 biosynthesis pathway (*panBCD* operon) in all *C. jejuni* ST50 and ST257 as well as in 32% of ST51 strains. The *panBCD* operon has been recently recognized as a marker of human pathogenic *C. jejuni* strains [17]. In addition, it seems to be a cattle-specific feature and the gain or loss of this locus was suggested as mechanism of rapid *C. jejuni* host switching from cattle to chickens [31]. Although chickens are generally considered as the ultimate vehicle responsible for transmission of *C. jejuni* to humans, then cattle may be an important source of strains contaminating the chicken production system, finally leading to human campylobacteriosis [17]. Furthermore, the *panBCD* operon is co-localized with the *bla*_*oxa-61*_ gene, that is also considered as a trait specific for strains causing infections in humans [17]. However, it is unclear whether this association is coincidental or somewhat linked with the agriculture niche and ability of *C. jejuni* to colonize ruminants. On the other hand, the study focused on zoonotic transmission of *C. jejuni* between birds, primates and livestock in US revealed that the presence of another beta-lactamase gene, *bla*_*oxa-184*,_ is specific for *C. jejuni* lineage limited to American crows and not associated with disease [32]. Since we detected *bla*_*oxa-184*_ in 68% of *C. jejuni* ST51 genomes, including one our carrier isolate (KF070), it is possible that their primary reservoir is not related to agriculture-associated animals or their transmission chain may differ from the used by strains possessing *bla*_*oxa-61*_. Moreover, since this group is characterized by the lowest number (mean = 11.9) of the 25 genes identified as determinants of *C. jejuni* strains causing infections in humans [17], then its pathogenic potential may be lower than the remaining ST51 or ST50 and ST257 strains.

The antimicrobial resistance patterns identified in our *C. jejuni* ST50, ST51 and ST257 strains, i.e. resistance to fluoroquinolones only or fluoroquinolones and tetracyclines, in general are consistent with the patterns observed in other *C. jejuni* genomes representing these STs. Since aminoglycoside resistance genes were recognized only in three genomes, and no macrolide or other antimicrobial resistance determinants were noted. These results are in line with epidemiological data from Poland (for details see an excellent paper by Wieczorek et al. [33]) showing low frequency of resistance to macrolides (from 0% to 3.3%) and aminoglycosides. On the other hand, level of resistance to fluoroquinolones either in our isolates (100%) and other Polish isolates ( > 90%) is critical on the background of European Union ( ~ 55%) [33–36]. Recently, some reports have shown that clonal spreading of *C. jejuni* fluoroquinolone resistant strains, for example of ST50 clone, is responsible for an emergence of resistance to fluoroquinolones in certain European countries [37]. However, its ubiquitous presence in phylogenetically unrelated STs in our country, indicates rather multiple independent mutation events as the primary cause. This situation is likely connected with a selective pressure associated with a broad use of fluoroquinolones in animal husbandry [33]. In contrast, resistance of *C. jejuni* to tetracyclines in Poland seems to be variable or locally dependent, and ranging from < 10% even to up 100% [33]. Although, resistance to tetracyclines in *C. jejuni* is generally considered as plasmid mediated [10], we noted various chromosomal localization of the *tetO* gene in 41.4% of genomes.

## Conclusions

*Campylobacter jejuni* ST50, ST51 and ST257 are among the top ten of STs isolated in Europe. However, WGS analysis revealed a notable diversity in occurrence of certain clinically relevant genes, serotypes and LOS classes even in *C. jejuni* strains representing the same STs, ST50 in particular. This observation deserves further clinical and epidemiological investigations as it might be related to risk associated with post-infectious development of GBS or MSF. In addition, the presence or lack of certain host-specific determinants, such as the vitamin B5 biosynthesis pathway or the *bla*_*oxa-184*_ gene may implicate various transmission chains or reservoirs of *C. jejuni* ST51 strains responsible for campylobacteriosis in northeastern Poland.

## Methods

Three predominant sequence types (STs), namely ST50 (CC-21), ST257 (CC-257) and ST51 (CC-443), were identified among *C. jejuni* isolates collected in our previous study on the etiology of acute diarrhea in northeastern Poland, ie. in Podlasie Province [30]. In detail, the *C. jejuni* strains were isolated from stool samples of children hospitalized with acute diarrhea in the Department of Pediatric Infectious Diseases of University Children's Hospital in Bialystok (Poland) [30]. The written informed consent from the children's parents or legal guardians for research studies were obtained, and the study was approved by the Bioethics Commission of the Medical University of Bialystok (Consent Number: R-I-002/31/2010 from 28 January 2010).

WGS of four *C. jejuni* isolates representing the most common STs: diarrheal isolates KF017 and KF042 (ST50), and KF045 (ST257) as well as KF070 strain (ST51) isolated from a control patient (without diarrhea), was performed on Ion PGM Machine (Life Technologies, USA) strictly followed the procedure developed in earlier study [38]. Sequences were assembled with Newbler v2.9 software (Roche, Germany) and deposited in the GenBank database under accession numbers: RDSQ00000000 (KF017), RDSP00000000 (KF042), RDSO00000000 (KF045), and RDSN00000000 (KF070).

In total, 1260 *C. jejuni* genomes collected in the PATRIC database (Pathosystems Resource Integration Center; accessed in November 2018) were screened for STs using mlst script; https://github.com/tseemann/mlst). In the next step, the genomes representing ST50 (n = 71), ST51 (n = 25) and ST257 (n = 20) (Additional file 1: Table S1) were annotated with Prokka v. 1.3.13 tool (with –usegenus and –genus Campylobacter parameters) [39] followed by pan-genome based phylogenetic analysis performed using Roary v. 3.11.2 software (with minimum percentage identity for blastp of 95% [-i parameter], and PRANK aligner v. 170427 to create a multiFASTA alignment of core genes [-e parameter]) [40, 41]. The Roary output, i.e. alignment of core genes, was used to built maximum-likelihood based on phylogenetic tree with bootstrap value of 500 iterations using RAxML tool v. 8.2.10 and the following parameters: [-m] GTRGAMMA [-p] 12,345 [-f] a [-x] 12,345 [-N] 500 [42].

Antibiotic resistance determinants were detected using ABRicate v0.8.5 software (https://github.com/tseemann/abricate) equipped with the CARD (Comprehensive Antibiotic Resistance Database) database [43]. In addition, PointFinder tool [44] was used to identify point mutations in *gyrA* (A70T, D85T, T86I, T86A, T86K, T86V, D90A, D90N, D90T, P104S), 23S rRNA (A2074G, A2074T, A2074C, A2075G), *cmeR* (A86G), *rplV* (A103C), and *rpsL* (K88E, K88R, K88Q) responsible for resistance to quinolones, macrolides and spectinomycin, respectively. Resistance patterns were verified by in vitro tests based on the disk diffusion method according to the EUCAST guidelines. The software tools were run with the default settings, except minimum DNA % coverage parameter [–mincov, default ‘0′] in ABRicate which was set to 30.

Virulence patterns were determined with ABRicate software using in-house created virulence gene database involving genes (n = 143) associated with motility, chemotaxis, adhesion, invasion, iron acquisition, capsule and LOS biosynthesis, general stress as well as T4SS and T6SS secretion systems (Additional file 1: Table S2). Additionally, we analyzed the prevalence of 25 genes identified as markers of human pathogenic *C. jejuni* strains [17]. Furthermore, *C. jejuni* serotypes were determined based on in silico multiplex PCR with primers described by Poly et al. [45] and simulate_pcr script [46]. Finally, *Campylobacter* spp. PubMLST database (https://pubmlst.org/campylobacter/; accessed in November 2018), was screened (58 179 records) for the prevalence of the STs under study.

NTSYSpc 2.2 (Exeter Software) and MEGA7 [47] were used to create dendrograms. Genome regions were compared and visualized with Easyfig v2.1 tool [48].

The Tukey's HSD for unequal N test (Spjotvolla/Stoline) was used to compare differences in average number of the virulence genes among the STs with Statistica v7 (StatSoft) software and a p-value 0.01 was considered statistically significant.

## Additional file

**Additional file 1:** **Table S1.** *C. jejuni* ST50, ST51 and ST257 genomes (n = 116) collected in the PATRIC genome database. **Table S2.** *C. jejuni* virulence genes (n = 143) under the study. **Table S3.** The twelve most common STs among *C. jejuni* genomes (n = 116) deposited in the PATRIC genome database. **Table S4.** The occurrence of virulence genes (n = 143) in *C. jejuni* ST50, ST51 and ST257 strains under the study. **Table S5.** The occurence of 25 genes recognized by Buchanan et al. [17] as markers of human pathogenic *C. jejuni* strains in genomes of C. jejuni ST50, ST51 and ST257. **Figure S1.** The distribution *C. jejuni* pan-genome genes and the number of isolates possessing them (visualized using roary_plots.py script). **Figure S2.** The frequency of *C. jejuni* genes versus the number of genomes (visualized using roary_plots.py script). **Figure S3.** The phylogenetic tree compared to a matrix with the presence and absence of *C. jejuni* core and accessory genes (visualized using roary_plots.py script). **Figure S4.** Similarity of *C. jejuni* 81-176 plasmid pTet (**A**) and *C. jejuni* CFSAN054107 plasmid pGMI16-002 (B) with *C. jejuni* strains under the study carrying *tetO* on plasmid contigs. **Figure S5.** UPGMA cluster tree illustrating identity of amino acid sequences of TetO variants detected in *C. jejuni* strains. The evolutionary distances were computed using the Poisson correction method, and are in the units of the number of amino acid substitutions per site. The tree was built in MEGA7 software. **Figure S6.** Chromosomal locations of the *tetO* gene in *C. jejuni* genomes under the study. **Figure S7.** Similarity of *C. jejuni* CFSAN054107 plasmid pGMI16-002 with *C. jejuni* genomes under the study carrying aminoglycoside resistance genes.

## Data Availability

The authors confirm that the data supporting the findings of this study are available within the article and/or its additional materials.
