# Supplementary material for: Whole-genome comparative analysis of Campylobacter jejuni strains isolated from patients with diarrhea in northeastern Poland
Source: Gut Pathog. 2019 Jun 19;11:32. doi: 10.1186/s13099-019-0313-x (PMC6582539; doi:10.1186/s13099-019-0313-x)
Supplement: Supplementary file 1 — Additional file 1: Table S1. C. jejuni ST50, ST51 and ST257 genomes (n = 116) collected in the PATRIC genome database. Table S2. C. jejuni virulence genes (n = 143) under the study. Table S3. The twelve most common STs among C. jejuni genomes (n = 116) deposited in the PATRIC genome database. Table S4. The occurrence of virulence genes (n = 143) in C. jejuni ST50, ST51 and ST257 strains under the study. Table S5. The occurence of 25 genes recognized by Buchanan et al. [17] as markers of human pathogenic C. jejuni strains in genomes of C. jejuni ST50, ST51 and ST257. Figure S1. The distribution C. jejuni pan-genome genes and the number of isolates possessing them (visualized using roary_plots.py script). Figure S2. The frequency of C. jejuni genes versus the number of genomes (visualized using roary_plots.py script). Figure S3. The phylogenetic tree compared to a matrix with the presence and absence of C. jejuni core and accessory genes (visualized using roary_plots.py script). Figure S4. Similarity of C. jejuni 81-176 plasmid pTet (A) and C. jejuni CFSAN054107 plasmid pGMI16-002 (B) with C. jejuni strains under the study carrying tetO on plasmid contigs. Figure S5. UPGMA cluster tree illustrating identity of amino acid sequences of TetO variants detected in C. jejuni strains. The evolutionary distances were computed using the Poisson correction method, and are in the units of the number of amino acid substitutions per site. The tree was built in MEGA7 software. Figure S6. Chromosomal locations of the tetO gene in C. jejuni genomes under the study. Figure S7. Similarity of C. jejuni CFSAN054107 plasmid pGMI16-002 with C. jejuni genomes under the study carrying aminoglycoside resistance genes. [file 13099_2019_313_MOESM1_ESM.pdf]

Whole-genome comparative analysis of *Campylobacter jejuni* strains isolated from patients with diarrhea in northeastern Poland

Krzysztof Fiedoruk[a,*], Tamara Daniluk[a], Dorota Rozkiewicz[b], Elzbieta Oldak[b], Suhanya Prasad[c], Izabela Swiecicka[d,e]

[a] Department of Microbiology, Medical University of Bialystok, Bialystok, Poland
[b] Department of Pediatric Infectious Diseases, Medical University of Bialystok, University Children's Hospital, Bialystok, Poland
[c] Department of Microbiological and Nanobiomedical Engineering, Medical University of Bialystok, Bialystok, Poland
[d] Department of Microbiology, University of Bialystok, Bialystok, Poland
[e] Laboratory of Applied Microbiology, University of Bialystok, Bialystok, Poland

Additional file 1

Table S1	*C. jejuni* ST50, ST51 and ST257 genomes (n=116) collected in the PATRIC genome database.
Table S2	*C. jejuni* virulence genes (n=143) under the study.
Table S3	The twelve most common STs among *C. jejuni* genomes (n=116) deposited in the PATRIC genome database.
Table S4	The occurrence of virulence genes (n=143) in *C. jejuni* ST50, ST51 and ST257 strains under the study.
Table S5	The occurence of 25 genes recognized by Buchanan et al. [17] as markers of human pathogenic *C. jejuni* strains in genomes of *C. jejuni* ST50, ST51 and ST257.
Figure S1	The distribution *C. jejuni* pan-genome genes and the number of isolates possessing them (visualized using roary_plots.py script).
Figure S2	The frequency of *C. jejuni* genes versus the number of genomes (visualized using roary_plots.py script).
Figure S3	The phylogenetic tree compared to a matrix with the presence and absence of *C. jejuni* core and accessory genes (visualized using roary_plots.py script).
Figure S4	Similarity of *C. jejuni* 81-176 plasmid pTet (A) and *C. jejuni* CFSAN054107 plasmid pGMI16-002 (B) with *C. jejuni* strains under the study carrying *tetO* on plasmid contigs.
Figure S5	UPGMA cluster tree illustrating identity of amino acid sequences of TetO variants detected in *C. jejuni* strains. The evolutionary distances were computed using the Poisson correction method, and are in the units of the number of amino acid substitutions per site. The tree was built in MEGA7 software.
Figure S6	Chromosomal locations of the *tetO* gene in *C. jejuni* genomes under the study.
Figure S7	Similarity of *C. jejuni* CFSAN054107 plasmid pGMI16-002 with *C. jejuni* genomes under the study carrying aminoglycoside resistance genes.

Table S1

C. jejuni ST50, ST51 and ST257 genomes (n=116) collected in the PATRIC genome database.

No.	Genome ID	Genome Name	Strain	ST	LOS class	Serotype	Isolation Source	Host Name	Isolation Country	Geographic Location	Collection Date	Contigs	Genome Length	GC Content	CDS	Genome Status
1	1440034.3	C. jejuni CVM 41912	CVM 41912	50	C	HS8c	Human	Human, Homo sapiens	United States	USA	2008	161	1711299	30.43	1918	WGS
2	1440029.3	C. jejuni CVM 41923	CVM 41923	50	C	HS8c	Human	Human, Homo sapiens	United States	USA	2009	71	1701940	30.42	1838	WGS
3	1201032.3	C. jejuni PT14	_	50	C	HS1c	_	_	_	_	_	1	1622145	30.60	1659	Complete
4	889255.3	C. jejuni 2008-831	2008-831	50	C	HS1c	_	_	_	_	_	120	1588304	30.50	1654	WGS
5	889250.3	C. jejuni 2008-1025	2008-1025	50	C	HS8c	_	_	_	_	_	139	1632888	30.40	1719	WGS
6	889228.3	C. jejuni 87330	87330	50	C	HS8c	_	Chicken, Gallus gallus	United States	USA	_	106	1597339	30.40	1650	WGS
7	32022.634	C. jejuni CFSAN065297	CFSAN065297	50	C	HS8c	feces	Human, Homo sapiens	Brazil	Brazil	2003	33	1701933	30.39	1823	WGS
8	32022.290	C. jejuni CJ504CC21	CJ504CC21	50	C	HS5c	Feces	Human, Homo sapiens	Finland	Finland	2006	26	1789612	30.36	1948	WGS
9	32022.288	C. jejuni CJ505CC21	CJ505CC21	50	C	HS5c	Feces	Human, Homo sapiens	Finland	Finland	2006	12	1717673	30.41	1839	WGS
10	32022.287	C. jejuni CJ506CC21	CJ506CC21	50	C	HS1c	Feces	Human, Homo sapiens	Finland	Finland	2006	19	1703992	30.35	1811	WGS
11	32022.286	C. jejuni CJ503CC21	CJ503CC21	50	C	HS1c	Feces	Human, Homo sapiens	Finland	Finland	2006	23	1690057	30.41	1791	WGS
12	32022.285	C. jejuni CJ500CC21	CJ500CC21	50	C	HS5c	Feces	Human, Homo sapiens	Finland	Finland	2006	35	1795173	30.34	1961	WGS
13	32022.284	C. jejuni CJ098CC21	CJ098CC21	50	C	HS1c	Blood	Human, Homo sapiens	Finland	Finland	2001	22	1626876	30.43	1712	WGS
14	32022.283	C. jejuni CJ502CC21	CJ502CC21	50	C	HS1c	Feces	Human, Homo sapiens	Finland	Finland	2006	24	1690290	30.41	1795	WGS
15	32022.282	C. jejuni CJ501CC21	CJ501CC21	50	C	HS1c	Feces	Human, Homo sapiens	Finland	Finland	2006	50	1794151	30.31	1967	WGS
16	32022.281	C. jejuni CJ006CC21	CJ006CC21	50	C	HS1c	Blood	Human, Homo sapiens	Finland	Finland	2001	25	1625765	30.42	1708	WGS
17	32022.280	C. jejuni CJ076CC21	CJ076CC21	50	C	HS1c	Blood	Human, Homo sapiens	Finland	Finland	2005	33	1802883	30.31	1968	WGS
18	32022.279	C. jejuni CJ069CC21	CJ069CC21	50	C	HS1c	Blood	Human, Homo sapiens	Finland	Finland	2000	20	1722019	30.35	1846	WGS
19	32022.276	C. jejuni CJ035CC21	CJ035CC21	50	C	HS1c	Blood	Human, Homo sapiens	Finland	Finland	2003	17	1625708	30.44	1700	WGS
20	32022.274	C. jejuni CJ003CC21	CJ003CC21	50	C	HS1c	Blood	Human, Homo sapiens	Finland	Finland	1998	20	1623955	30.43	1699	WGS
21	197.6758	C. jejuni BCW_5141	BCW_5141	50	C	HS8c	_	Human, Homo sapiens	_	_	_	73	1701529	30.58	1805	WGS
22	197.6746	C. jejuni BCW_5123	BCW_5123	50	C	HS8c	_	Human, Homo sapiens	_	_	_	60	1722506	30.43	1841	WGS
23	197.6704	C. jejuni BCW_6919	BCW_6919	50	C	HS8c	feces	Ovine	_	_	_	45	1723669	30.63	1801	WGS
24	197.5328	C. jejuni WP2202	WP2202	50	C	HS8c	gizzard	Chicken, Gallus gallus	United States	USA	2009	2	1801450	30.30	1906	Complete
25	197.5327	C. jejuni OD267	OD267	50	C	HS8c	liver	Chicken, Gallus gallus	United States	USA	2009	3	1826322	30.20	1956	Complete
26	197.5228	C. jejuni	Campylobacter jejuni	50	C	HS1c	Madrid	Chicken, Gallus gallus	_	_	2010	61	1614704	30.48	1720	WGS
27	197.5184	C. jejuni	Campylobacter jejuni	50	C	HS1c	Madrid	_	_	_	2010	65	1657224	30.46	1772	WGS
28	197.5165	C. jejuni RM1285	RM1285	50	C	HS1c	Exudate from chicken breast purchased at a retail location	Chicken, Gallus gallus	United States	USA: Albany CA	1997	1	1635803	30.54	1692	Complete
29	197.5133	C. jejuni OXC6459	OXC6459	50	C	HS8c	faeces	Human, Homo sapiens	United Kingdom	United Kingdom	2011	21	1666090	30.38	1744	WGS
30	197.5113	C. jejuni OXC6286	OXC6286	50	C	HS8c	faeces	Human, Homo sapiens	United Kingdom	United Kingdom	2011	17	1676099	30.34	1746	WGS
31	197.5110	C. jejuni OXC6489	OXC6489	50	C	HS1c	faeces	Human, Homo sapiens	United Kingdom	United Kingdom	2011	14	1627281	30.42	1692	WGS
32	197.5099	C. jejuni OXC6277	OXC6277	50	C	HS10	faeces	Human, Homo sapiens	United Kingdom	United Kingdom	2011	12	1657150	30.46	1744	WGS
33	197.5092	C. jejuni OXC6600	OXC6600	50	C	HS8c	faeces	Human, Homo sapiens	United Kingdom	United Kingdom	2011	18	1661585	30.38	1733	WGS
34	197.5076	C. jejuni OXC6524	OXC6524	50	C	HS8c	faeces	Human, Homo sapiens	United Kingdom	United Kingdom	2011	16	1666245	30.37	1736	WGS
35	197.5074	C. jejuni OXC6457	OXC6457	50	C	HS1c	faeces	Human, Homo sapiens	United Kingdom	United Kingdom	2011	20	1730021	30.35	1852	WGS
36	197.5072	C. jejuni OXC6613	OXC6613	50	C	HS1c	faeces	Human, Homo sapiens	United Kingdom	United Kingdom	2011	23	1726021	30.36	1827	WGS
37	197.5038	C. jejuni OXC6565	OXC6565	50	C	HS8c	faeces	Human, Homo sapiens	United Kingdom	United Kingdom	2011	20	1662824	30.38	1737	WGS
38	197.5035	C. jejuni OXC6292	OXC6292	50	C	HS8c	faeces	Human, Homo sapiens	United Kingdom	United Kingdom	2011	19	1675483	30.34	1748	WGS
39	197.5026	C. jejuni OXC6531	OXC6531	50	C	HS1c	faeces	Human, Homo sapiens	United Kingdom	United Kingdom	2011	23	1667473	30.46	1763	WGS
40	197.5020	C. jejuni OXC6571	OXC6571	50	C	HS8c	faeces	Human, Homo sapiens	United Kingdom	United Kingdom	2011	21	1676845	30.34	1747	WGS
41	197.5004	C. jejuni OXC6461	OXC6461	50	C	HS8c	faeces	Human, Homo sapiens	United Kingdom	United Kingdom	2011	15	1665638	30.37	1738	WGS
42	197.5003	C. jejuni OXC6493	OXC6493	50	C	HS10	faeces	Human, Homo sapiens	United Kingdom	United Kingdom	2011	17	1664469	30.45	1756	WGS
43	197.5001	C. jejuni OXC6530	OXC6530	50	C	HS8c	faeces	Human, Homo sapiens	United Kingdom	United Kingdom	2011	17	1666036	30.37	1739	WGS
44	197.4983	C. jejuni OXC6331	OXC6331	50	C	HS8c	faeces	Human, Homo sapiens	United Kingdom	United Kingdom	2011	17	1676876	30.38	1749	WGS
45	197.4952	C. jejuni OXC6347	OXC6347	50	C	HS8c	faeces	Human, Homo sapiens	United Kingdom	United Kingdom	2011	17	1677216	30.38	1742	WGS
46	197.4950	C. jejuni OXC6502	OXC6502	50	C	HS10	faeces	Human, Homo sapiens	United Kingdom	United Kingdom	2011	17	1659343	30.45	1749	WGS
47	197.4933	C. jejuni OXC6449	OXC6449	50	C	HS1c	faeces	Human, Homo sapiens	United Kingdom	United Kingdom	2011	13	1629571	30.48	1680	WGS
48	197.4925	C. jejuni OXC6615	OXC6615	50	C	HS8c	faeces	Human, Homo sapiens	United Kingdom	United Kingdom	2011	18	1640255	30.41	1700	WGS
49	197.4910	C. jejuni OXC6636	OXC6636	50	C	HS8c	faeces	Human, Homo sapiens	United Kingdom	United Kingdom	2011	17	1666281	30.37	1740	WGS
50	197.4908	C. jejuni OXC6326	OXC6326	50	C	HS1c	faeces	Human, Homo sapiens	United Kingdom	United Kingdom	2011	16	1662776	30.45	1741	WGS
51	197.4901	C. jejuni OXC6590	OXC6590	50	C	HS8c	faeces	Human, Homo sapiens	United Kingdom	United Kingdom	2011	15	1667206	30.37	1739	WGS
52	197.4871	C. jejuni OXC6289	OXC6289	50	C	HS8c	faeces	Human, Homo sapiens	United Kingdom	United Kingdom	2011	20	1700941	30.34	1792	WGS
53	197.4838	C. jejuni OXC6598	OXC6598	50	C	HS8c	faeces	Human, Homo sapiens	United Kingdom	United Kingdom	2011	16	1662997	30.39	1736	WGS
54	197.4824	C. jejuni OXC6266	OXC6266	50	C	HS8c	faeces	Human, Homo sapiens	United Kingdom	United Kingdom	2011	20	1675962	30.34	1747	WGS
55	197.4816	C. jejuni OXC6527	OXC6527	50	C	HS8c	faeces	Human, Homo sapiens	United Kingdom	United Kingdom	2011	16	1666553	30.37	1734	WGS
56	197.4814	C. jejuni OXC6543	OXC6543	50	C	HS8c	faeces	Human, Homo sapiens	United Kingdom	United Kingdom	2011	22	1666364	30.37	1742	WGS
57	197.4810	C. jejuni	_	50	A	HS2	_	_	_	_	_	73	1675779	30.64	1775	WGS
58	197.4809	C. jejuni	_	50	A	HS2	_	_	_	_	_	60	1684609	30.70	1748	WGS
59	197.4807	C. jejuni	_	50	A	HS2	_	_	_	_	_	54	1663792	30.58	1741	WGS
60	197.4806	C. jejuni	_	50	A	HS2	_	_	_	_	_	48	1682333	30.68	1748	WGS
61	197.4805	C. jejuni	_	50	A	HS2	_	_	_	_	_	57	1668481	30.62	1742	WGS
62	197.4804	C. jejuni	_	50	A	HS2	_	_	_	_	_	50	1675030	30.66	1741	WGS
63	197.13332	C. jejuni NCTC13357	NCTC13357	50	C	HS8c	_	_	_	_	_	3	1812524	30.30	1913	WGS
64	197.13052	C. jejuni CAMSA2104	CAMSA2104	50	C	HS8c	_	broiler chicken	Denmark	Denmark: Copenhagen	2009	63	1714008	30.41	1852	WGS
65	197.13041	C. jejuni R4-B2-08	R4-B2-08	50	C	HS1c	_	_	United States	USA	1983	321	1745148	30.78	2019	WGS
66	197.11580	C. jejuni RM3412	RM3412	50	C	HS8c	feces	Human, Homo sapiens	Canada	Canada	1980	1698	1612440	30.82	1700	WGS
67	197.6730	C. jejuni BCW_4731	BCW_4731	50	C	HS1c	_	_	_	_	_	32	1698127	30.59	1779	WGS
68	197.4808	C. jejuni	_	50	C	HS1c	_	_	_	_	_	48	1870255	30.58	1966	WGS
69	197.11657	C. jejuni isolate_2	isolate_2	50	C	HS8c	retail chicken	Chicken, Gallus gallus	Canada	Canada	2008	134	1819297	30.16	2024	WGS
70	197.14642	C. jejuni KF017	KF017	50	C	HS8c	faeces	Human, Homo sapiens	Poland	Poland	2010	30	1669263	30.42	1726	WGS
71	197.14644	C. jejuni KF042	KF042	50	C	HS8c	faeces	Human, Homo sapiens	Poland	Poland	2010	50	1668012	30.42	1743	WGS
72	1321759.3	C. jejuni HB-CJGB-ZX	HB-CJGB-ZX	51	E	HS37	one Guillain-Barr syndrome patient	Human, Homo sapiens	China	China	_	11	1680820	30.31	1779	WGS
73	1316917.3	C. jejuni HB-CJGB-LL	HB-CJGB-LL	51	E	HS37	stool specimen from a Guillain-Barre syndrome patient	Human, Homo sapiens	China	China	1993	8	1680912	30.24	1796	WGS
74	32022.610	C. jejuni CFSAN065333	CFSAN065333	51	E	HS37	feces	Chicken, Gallus gallus	Brazil	Brazil	2004	21	1701738	30.32	1840	WGS
75	32022.609	C. jejuni CFSAN065334	CFSAN065334	51	E	HS37	feces	Chicken, Gallus gallus	Brazil	Brazil	2004	22	1700593	30.31	1840	WGS
76	32022.608	C. jejuni CFSAN065335	CFSAN065335	51	E	HS37	feces	Chicken, Gallus gallus	Brazil	Brazil	2004	21	1701288	30.32	1844	WGS
77	197.7921	C. jejuni CDPHFDLB-F12M00554-a3	CDPHFDLB-F12M00554-a3	51	E	HS37	Water	_	United States	USA:CA	2012-05	17	1715031	30.30	1844	WGS
78	197.7920	C. jejuni CDPHFDLB-F12M00554-a2	CDPHFDLB-F12M00554-a2	51	E	HS37	Water	_	United States	USA:CA	2012-05	27	1715985	30.31	1851	WGS
79	197.7745	C. jejuni BCW_5167	BCW_5167	51	E	HS37	_	Human, Homo sapiens	_	_	_	32	1715657	30.53	1802	WGS
80	197.5185	C. jejuni	Campylobacter jejuni	51	E	HS37	Madrid	Chicken, Gallus gallus	Madrid	Madrid	2010	93	1719159	30.27	1901	WGS
81	197.5174	C. jejuni	Campylobacter jejuni	51	E	HS37	Madrid	Chicken, Gallus gallus	Madrid	Madrid	2010	186	1654423	30.43	1870	WGS
82	197.5167	C. jejuni	Campylobacter jejuni	51	E	HS37	Madrid	Chicken, Gallus gallus	Madrid	Madrid	2010	88	1635512	30.33	1803	WGS
83	197.5137	C. jejuni OXC6274	OXC6274	51	E	HS37	faeces	Human, Homo sapiens	United Kingdom	United Kingdom	2011	17	1641313	30.35	1735	WGS
84	197.5088	C. jejuni OXC6396	OXC6396	51	E	HS37	faeces	Human, Homo sapiens	United Kingdom	United Kingdom	2011	15	1677641	30.29	1785	WGS
85	197.5069	C. jejuni OXC6357	OXC6357	51	E	HS37	faeces	Human, Homo sapiens	United Kingdom	United Kingdom	2011	14	1638988	30.35	1733	WGS
86	197.5043	C. jejuni OXC6316	OXC6316	51	E	HS37	faeces	Human, Homo sapiens	United Kingdom	United Kingdom	2011	10	1708669	30.28	1820	WGS
87	197.5010	C. jejuni OXC6503	OXC6503	51	E	HS37	faeces	Human, Homo sapiens	United Kingdom	United Kingdom	2011	17	1668989	30.33	1786	WGS
88	197.4994	C. jejuni OXC6342	OXC6342	51	E	HS37	faeces	Human, Homo sapiens	United Kingdom	United Kingdom	2011	13	1664818	30.33	1784	WGS
89	197.4975	C. jejuni OXC6311	OXC6311	51	E	HS37	faeces	Human, Homo sapiens	United Kingdom	United Kingdom	2011	19	1701008	30.35	1836	WGS
90	197.4956	C. jejuni OXC6440	OXC6440	51	E	HS37	faeces	Human, Homo sapiens	United Kingdom	United Kingdom	2011	12	1715467	30.32	1826	WGS
91	197.4870	C. jejuni OXC6620	OXC6620	51	E	HS37	faeces	Human, Homo sapiens	United Kingdom	United Kingdom	2011	12	1676039	30.36	1787	WGS
92	197.4867	C. jejuni OXC6366	OXC6366	51	E	HS37	faeces	Human, Homo sapiens	United Kingdom	United Kingdom	2011	13	1710149	30.28	1823	WGS
93	197.4864	C. jejuni OXC6302	OXC6302	51	E	HS37	faeces	Human, Homo sapiens	United Kingdom	United Kingdom	2011	15	1638081	30.36	1733	WGS
94	197.4827	C. jejuni OXC6609	OXC6609	51	E	HS37	faeces	Human, Homo sapiens	United Kingdom	United Kingdom	2011	14	1639228	30.35	1733	WGS
95	197.11671	C. jejuni isolate_U1	isolate_U1	51	E	HS37	retail chicken	_	Canada	Canada	2008	56	1627350	30.32	1744	WGS
96	197.14645	C. jejuni KF070	KF070	51	E	HS37	faeces	Human, Homo sapiens	Poland	Poland	2010	20	1662863	30.33	1716	WGS
97	1248402.4	C. jejuni BIGS0022	BIGS0022	257	D	HS11	Chicken offal or meat	Chicken, Gallus gallus	United Kingdom	United Kingdom	2001	169	1573736	30.85	1760	WGS
98	197.7960	C. jejuni BCW_5160	BCW_5160	257	D	HS11	_	Human, Homo sapiens	_	_	_	54	1824739	30.41	1964	WGS
99	197.6740	C. jejuni BCW_4747	BCW_4747	257	D	HS11	_	_	_	_	_	22	1718223	30.38	1812	WGS
100	197.5258	C. jejuni n/a	_	257	D	HS11	farm environment	Poultry House, Faecal	United Kingdom	_	2013	37	1648287	30.34	1760	WGS
101	197.5257	C. jejuni n/a	_	257	D	HS11	farm environment	Poultry Environment, bootsock	United Kingdom	_	2013	29	1643261	30.34	1754	WGS
102	197.5106	C. jejuni OXC6375	OXC6375	257	D	HS11	faeces	Human, Homo sapiens	United Kingdom	United Kingdom	2011	13	1731121	30.29	1857	WGS
103	197.5065	C. jejuni OXC6344	OXC6344	257	D	HS11	faeces	Human, Homo sapiens	United Kingdom	United Kingdom	2011	17	1681158	30.33	1801	WGS
104	197.5050	C. jejuni OXC6407	OXC6407	257	D	HS11	faeces	Human, Homo sapiens	United Kingdom	United Kingdom	2011	11	1606321	30.44	1706	WGS
105	197.5036	C. jejuni OXC6612	OXC6612	257	D	HS11	faeces	Human, Homo sapiens	United Kingdom	United Kingdom	2011	16	1731524	30.29	1862	WGS
106	197.5025	C. jejuni OXC6390	OXC6390	257	D	HS11	faeces	Human, Homo sapiens	United Kingdom	United Kingdom	2011	18	1681499	30.33	1804	WGS
107	197.5015	C. jejuni OXC6290	OXC6290	257	D	HS11	faeces	Human, Homo sapiens	United Kingdom	United Kingdom	2011	11	1695865	30.27	1806	WGS
108	197.5013	C. jejuni OXC6412	OXC6412	257	D	HS11	faeces	Human, Homo sapiens	United Kingdom	United Kingdom	2011	17	1693360	30.25	1818	WGS
109	197.5006	C. jejuni OXC6491	OXC6491	257	D	HS11	faeces	Human, Homo sapiens	United Kingdom	United Kingdom	2011	13	1724497	30.25	1856	WGS
110	197.4991	C. jejuni OXC6250	OXC6250	257	D	HS11	faeces	Human, Homo sapiens	United Kingdom	United Kingdom	2011	11	1686542	30.32	1782	WGS
111	197.4976	C. jejuni OXC6450	OXC6450	257	D	HS11	faeces	Human, Homo sapiens	United Kingdom	United Kingdom	2011	18	1681761	30.28	1786	WGS
112	197.4973	C. jejuni OXC6401	OXC6401	257	D	HS11	faeces	Human, Homo sapiens	United Kingdom	United Kingdom	2011	11	1643371	30.34	1742	WGS
113	197.4930	C. jejuni OXC6362	OXC6362	257	D	HS11	faeces	Human, Homo sapiens	United Kingdom	United Kingdom	2011	15	1711126	30.29	1840	WGS
114	197.4860	C. jejuni OXC6254	OXC6254	257	D	HS11	faeces	Human, Homo sapiens	United Kingdom	United Kingdom	2011	16	1711245	30.29	1841	WGS
115	197.4839	C. jejuni OXC6583	OXC6583	257	D	HS11	faeces	Human, Homo sapiens	United Kingdom	United Kingdom	2011	17	1729681	30.29	1850	WGS
116	197.14643	C. jejuni KF045	KF045	257	D	HS11	faeces	Human, Homo sapiens	Poland	Poland	2010	36	1671769	30.43	1774	WGS

Table S2
C. jejuni virulence genes (n=143) under the study.

Function	Gene	Source
capsule biosynthesis	*cysC*	*Campylobacter jejuni* subsp. *jejuni* NCTC 11168-BN148 (Accession number HE978252)
	fcl	
	glf	
	gmhA2	
	hddA	
	hddC1	
	hddC2	
	kfiD	
	kpsC	
	kpsD	
	kpsE	
	kpsF	
	kpsM	
	kpsS	
	kpsT	
	rfbC	
	cj1416c	
	cj1421c	
	cj1422c	
	cj1426c	
	cj1427c	
	cj1429c	
	cj1432c	
	cj1433c	
	cj1434c	
	cj1435c	
	cj1436c	
	cj1437c	
	cj1438c	
	cj1440c	
	cj1442c	
motility and chemotaxis	*acfB*	*Campylobacter jejuni* subsp. *jejuni* NCTC 11168-BN148 (Accession number HE978252)
	cetA	
	cetB	
	cheA	
	cheB	
	cheR	
	cheV	
	cheW	
	cheY	
	cj0371	
	cj1321	
	cj1323	
	cj1324	
	cj1325	
	flaA	
	flaB	
	flaC	
	flaD	
	flaG	
	flgB	
	flgC	
	flgD	
	flgE1	
	flgE2	
	flgF	
	flgG	
	flgH	
	flgI	
	flgK	
	flhA	
	flhB	
	flhF	
	fliA	
	fliD	
	fliE	
	fliF	
	fliG	
	fliH	
	fliI	
	fliK	
	fliL	
	fliM	
	fliN	
	fliP	
	fliQ	
	fliR	
	fliS	
	fliY	
	luxS	
	motA	
	motB	
	pflA	
	pseG	
	pseH	
	ptmA	
	ptmB	
	rpon	
	tlp1	
	tlp10	
adhesion	*cadF*	*Campylobacter jejuni* subsp. *jejuni* NCTC 11168-BN148 (Accession number HE978252)
	jlpA	
	peb1A	
	peb3	
	pldA	
	porA	
	ggt	*Campylobacter jejuni* subsp. *jejuni* 81-176 (Accession number CP000538)
stress response	*cj0358*	*Campylobacter jejuni* subsp. *jejuni* NCTC 11168-BN148 (Accession number HE978252)
	cj1371	
	ahpC	
	cj0012c	
	cj0020c	
	dnaJ	
	katA	
	spoT	
	tpx	
	csrA	*Campylobacter jejuni* subsp. *jejuni* 81-176 (Accession number CP000538)
iron acquisition	*ceuE*	*Campylobacter jejuni* subsp. *jejuni* NCTC 11168-BN148 (Accession number HE978252)
	cfrA	
	chuA	
	cj0178	
toxin	*cdtA*	*Campylobacter jejuni* subsp. *jejuni* NCTC 11168-BN148 (Accession number HE978252)
	cdtB	
	cdtC	
type IV secretion systems (T4SS)	*virD4*	*Campylobacter jejuni* subsp. *jejuni* 81-176 plasmid pVir (Accession number CP000550)
	virB9	
	virB8	
	VirB7	
	virB4	
	virB11	
	virB10	
type VI secretion systems (T6SS)	*tssA*	*Campylobacter jejuni* strain 108 (Accession number JX436460)
	tssB	
	tssC	
	tssD	
	tssE	
	tssF	
	tssG	
	tagH	
	tssI	
	tssJ	
	tssK	
	tssL	
	tssM	
LOS class	A	Accession number AF130984
	B	Accession number AF401529
	C	Accession number AY044156
	D	Accession number AF130984
	E	Accession number AF343914

Table S3
The twelve most common STs among *C. jejuni* genomes (n=116) deposited in the PATRIC genome database.

ST	Number of ST	% of the total STs*
50	67	5.3
21	66	5.2
45	60	4.8
677	48	3.8
48	31	2.5
922	31	2.5
8	29	2.3
353	28	2.2
22	25	2.0
51	24	1.9
61	21	1.7
257	19	1.5

* 324 is the total number of distinct STs, profiles without ascribed ST were excluded

Table S4

The occurrence of virulence genes (n=143) in *C. jejuni* ST50, ST51 and ST257 strains under the study.

Genome ID	ST	Number of genes	cj1321_motility	cj1323_motility	Cj1416c_capsule	cysC_capsule	ciaB_invasion	pseH_motility	CheY_chemotaxis	Cj0358_stress_response	Cj0371_motility	Cj1371_stress_response	acfB_chemotaxis	ahpC_stress_response	cdtA_toxin	cdtB_toxin	cdtC_toxin	cetA_chemotaxis	ceuE_iron_acquisition	cfrA_iron_acquisition	cheA_chemotaxis	cheB_chemotaxis	cheV_chemotaxis	cheW_chemotaxis	cj0012c_stress_response	cj0020c_stress_response	cj0178_iron_acquisition	csrA_stress_response	dnaJ_stress_response	flaD_motility	flaG_motility	flgB_motility	flgC_motility	flgD_motility	flgE1_motility	flgF_motility	flgG_motility	flgH_motility	flgI_motility	flgK_motility	flhF_motility	fliA_motility	fliD_motility	fliE_motility	fliF_motility	fliG_motility	fliH_motility	fliI_motility	fliL_motility	fliM_motility	fliN_motility	fliR_motility	fliS_motility	fliY_motility	htrA_invasion	iamA_invasion	jlpA_adhesion	katA_stress_response	kpsD_capsule	kpsE_capsule	kpsF_capsule	kpsM_capsule	kpsT_capsule	luxS_chemotaxis	motA_motility	motB_motility	peb1A_adhesion	peb3_adhesion	pldA_adhesion	porA_adhesion	pseG_motility	ptmA_motility	ptmB_motility	rpon_motility	spoT_stress_response	tlp10_chemotaxis	tlp1_chemotaxis	tpx_stress_response	cetB_chemotaxis	kpsC_capsule	kpsS_capsule	pflA_motility	cadF_adhesion	ciaC_invasion	cheR_chemotaxis	chuA_iron_acquisition	fliQ_invasion	fliK_motility	cj1324_motility	cj1325_motility	LOS_class_C	gmhA2_capsule	hddA_capsule	hddC1_capsule	rfbC_capsule	Cj1429c_capsule	flaA_motility	flaB_motility	LOS_class_E	flgE2_motility	TagH_VasC_t6ss	TssG_VasB_t6ss	LOS_class_D	Cj1421c_capsule	Cj1422c_capsule	Cj1427c_capsule	Cj1432c_capsule	Cj1433c_capsule	Cj1435c_capsule	Cj1436c_capsule	Cj1437c_capsule	Cj1440c_capsule	glf_capsule	hddC2_capsule	kfiD_capsule	LOS_class_A	Cj1438c_capsule	Cj1442c_capsule	fcl_capsule	Cj1426c_capsule	TssA_VasJ_t6ss	TssB_VipA_t6ss	TssC_VipB_t6ss	TssD_Hcp_t6ss	TssE_t6ss	TssF_VasA_t6ss	TssJ_VasD_t6ss	TssK_VasE_t6ss	TssL_VasF_t6ss	TssM_VasK_t6ss	TssI_VgrG_t6ss	Cjp54_t4ss	virB10_t4ss	virB11_t4ss	virB4_t4ss	virB8_t4ss	virB9_t4ss	virD4_t4ss	flaC_motility	flhB_motility	Cj1434c_capsule	LOS_class_B	flhA_motility	fliP_motility	ggt_adhesion
197.4908	ST50	89	1	1	1	1	1	1	1	1	1	1	1	1	1	1	1	1	1	1	1	1	1	1	1	1	1	1	1	1	1	1	1	1	1	1	1	1	1	1	1	1	1	1	1	1	1	1	1	1	1	1	1	1	1	1	1	1	1	1	1	1	1	1	1	1	1	1	1	1	1	1	1	1	1	1	1	1	1	1	1	1	1	1	1	1	1	1	1	0	0	0	0	0	0	0	0	0	0	0	0	0	0	0	0	0	0	0	0	0	0	0	0	0	0	0	0	0	0	0	0	0	0	0	0	0	0	0	0	0	0	0	0	0	0	0	0	0	0	0	0	0
197.5074	ST50	89	1	1	1	1	1	1	1	1	1	1	1	1	1	1	1	1	1	1	1	1	1	1	1	1	1	1	1	1	1	1	1	1	1	1	1	1	1	1	1	1	1	1	1	1	1	1	1	1	1	1	1	1	1	1	1	1	1	1	1	1	1	1	1	1	1	1	1	1	1	1	1	1	1	1	1	1	1	1	1	1	1	1	1	1	1	1	1	0	0	0	0	0	0	0	0	0	0	0	0	0	0	0	0	0	0	0	0	0	0	0	0	0	0	0	0	0	0	0	0	0	0	0	0	0	0	0	0	0	0	0	0	0	0	0	0	0	0	0	0	0
197.5110	ST50	89	1	1	1	1	1	1	1	1	1	1	1	1	1	1	1	1	1	1	1	1	1	1	1	1	1	1	1	1	1	1	1	1	1	1	1	1	1	1	1	1	1	1	1	1	1	1	1	1	1	1	1	1	1	1	1	1	1	1	1	1	1	1	1	1	1	1	1	1	1	1	1	1	1	1	1	1	1	1	1	1	1	1	1	1	1	1	1	0	0	0	0	0	0	0	0	0	0	0	0	0	0	0	0	0	0	0	0	0	0	0	0	0	0	0	0	0	0	0	0	0	0	0	0	0	0	0	0	0	0	0	0	0	0	0	0	0	0	0	0	0
32022.287	ST50	89	1	1	1	1	1	1	1	1	1	1	1	1	1	1	1	1	1	1	1	1	1	1	1	1	1	1	1	1	1	1	1	1	1	1	1	1	1	1	1	1	1	1	1	1	1	1	1	1	1	1	1	1	1	1	1	1	1	1	1	1	1	1	1	1	1	1	1	1	1	1	1	1	1	1	1	1	1	1	1	1	1	1	1	1	1	1	1	0	0	0	0	0	1	0	0	0	0	0	0	0	0	0	0	0	0	0	0	0	0	0	0	0	0	0	0	0	0	0	0	0	0	0	0	0	0	0	0	0	0	0	0	0	0	0	0	0	0	0	0	0
197.13041	ST50	90	1	1	1	1	1	1	1	1	1	1	1	1	1	1	1	1	1	1	1	1	1	1	1	1	1	1	1	1	1	1	1	1	1	1	1	1	1	1	1	1	1	1	1	1	1	1	1	1	1	1	1	1	1	1	1	1	1	1	1	1	1	1	1	1	1	1	1	1	1	1	1	1	1	1	1	1	1	1	1	1	1	1	1	1	1	1	1	0	0	0	0	0	1	0	0	0	0	0	0	0	0	0	0	0	0	0	0	0	0	0	0	0	0	0	0	0	0	0	0	0	0	0	0	0	0	0	0	0	0	0	0	0	0	0	0	0	0	0	0	0
32022.281	ST50	90	1	1	1	1	1	1	1	1	1	1	1	1	1	1	1	1	1	1	1	1	1	1	1	1	1	1	1	1	1	1	1	1	1	1	1	1	1	1	1	1	1	1	1	1	1	1	1	1	1	1	1	1	1	1	1	1	1	1	1	1	1	1	1	1	1	1	1	1	1	1	1	1	1	1	1	1	1	1	1	1	1	1	1	1	1	1	1	0	0	0	0	0	1	0	0	0	0	0	0	0	0	0	0	0	0	0	0	0	0	0	0	0	0	0	0	0	0	0	0	0	0	0	0	0	0	0	0	0	0	0	0	0	0	0	0	0	0	0	0	0
32022.284	ST50	90	1	1	1	1	1	1	1	1	1	1	1	1	1	1	1	1	1	1	1	1	1	1	1	1	1	1	1	1	1	1	1	1	1	1	1	1	1	1	1	1	1	1	1	1	1	1	1	1	1	1	1	1	1	1	1	1	1	1	1	1	1	1	1	1	1	1	1	1	1	1	1	1	1	1	1	1	1	1	1	1	1	1	1	1	1	1	1	0	0	0	0	0	1	1	0	0	0	0	0	0	0	0	0	0	0	0	0	0	0	0	0	0	0	0	0	0	0	0	0	0	0	0	0	0	0	0	0	0	0	0	0	0	0	0	0	0	0	0	0	0
1201032.3	ST50	91	1	1	1	1	1	1	1	1	1	1	1	1	1	1	1	1	1	1	1	1	1	1	1	1	1	1	1	1	1	1	1	1	1	1	1	1	1	1	1	1	1	1	1	1	1	1	1	1	1	1	1	1	1	1	1	1	1	1	1	1	1	1	1	1	1	1	1	1	1	1	1	1	1	1	1	1	1	1	1	1	1	1	1	1	1	1	1	0	0	0	0	0	1	1	0	0	0	0	0	0	0	0	0	0	0	0	0	0	0	0	0	0	0	0	0	0	0	0	0	0	0	0	0	0	0	0	0	0	0	0	0	0	0	0	0	0	0	0	0	0
197.4808	ST50	91	1	1	1	1	1	1	1	1	1	1	1	1	1	1	1	1	1	1	1	1	1	1	1	1	1	1	1	1	1	1	1	1	1	1	1	1	1	1	1	1	1	1	1	1	1	1	1	1	1	1	1	1	1	1	1	1	1	1	1	1	1	1	1	1	1	1	1	1	1	1	1	1	1	1	1	1	1	1	1	1	1	1	1	1	1	1	1	0	0	0	0	0	1	1	0	0	0	0	0	0	0	0	0	0	0	0	0	0	0	0	0	0	0	0	0	0	0	0	0	0	0	0	0	0	0	0	0	0	0	0	0	0	0	0	0	0	0	0	0	0
197.4933	ST50	91	1	1	1	1	1	1	1	1	1	1	1	1	1	1	1	1	1	1	1	1	1	1	1	1	1	1	1	1	1	1	1	1	1	1	1	1	1	1	1	1	1	1	1	1	1	1	1	1	1	1	1	1	1	1	1	1	1	1	1	1	1	1	1	1	1	1	1	1	1	1	1	1	1	1	1	1	1	1	1	1	1	1	1	1	1	1	1	0	0	0	0	0	1	1	0	0	0	0	0	0	0	0	0	0	0	0	0	0	0	0	0	0	0	0	0	0	0	0	0	0	0	0	0	0	0	0	0	0	0	0	0	0	0	0	0	0	0	0	0	0
197.5165	ST50	91	1	1	1	1	1	1	1	1	1	1	1	1	1	1	1	1	1	1	1	1	1	1	1	1	1	1	1	1	1	1	1	1	1	1	1	1	1	1	1	1	1	1	1	1	1	1	1	1	1	1	1	1	1	1	1	1	1	1	1	1	1	1	1	1	1	1	1	1	1	1	1	1	1	1	1	1	1	1	1	1	1	1	1	1	1	1	1	0	0	0	0	0	1	1	0	0	0	0	0	0	0	0	0	0	0	0	0	0	0	0	0	0	0	0	0	0	0	0	0	0	0	0	0	0	0	0	0	0	0	0	0	0	0	0	0	0	0	0	0	0
197.6730	ST50	91	1	1	1	1	1	1	1	1	1	1	1	1	1	1	1	1	1	1	1	1	1	1	1	1	1	1	1	1	1	1	1	1	1	1	1	1	1	1	1	1	1	1	1	1	1	1	1	1	1	1	1	1	1	1	1	1	1	1	1	1	1	1	1	1	1	1	1	1	1	1	1	1	1	1	1	1	1	1	1	1	1	1	1	1	1	1	1	0	0	0	0	0	1	1	0	0	0	0	0	0	0	0	0	0	0	0	0	0	0	0	0	0	0	0	0	0	0	0	0	0	0	0	0	0	0	0	0	0	0	0	0	0	0	0	0	0	0	0	0	0
32022.274	ST50	91	1	1	1	1	1	1	1	1	1	1	1	1	1	1	1	1	1	1	1	1	1	1	1	1	1	1	1	1	1	1	1	1	1	1	1	1	1	1	1	1	1	1	1	1	1	1	1	1	1	1	1	1	1	1	1	1	1	1	1	1	1	1	1	1	1	1	1	1	1	1	1	1	1	1	1	1	1	1	1	1	1	1	1	1	1	1	1	0	0	0	0	0	1	1	0	0	0	0	0	0	0	0	0	0	0	0	0	0	0	0	0	0	0	0	0	0	0	0	0	0	0	0	0	0	0	0	0	0	0	0	0	0	0	0	0	0	0	0	0	0
32022.276	ST50	91	1	1	1	1	1	1	1	1	1	1	1	1	1	1	1	1	1	1	1	1	1	1	1	1	1	1	1	1	1	1	1	1	1	1	1	1	1	1	1	1	1	1	1	1	1	1	1	1	1	1	1	1	1	1	1	1	1	1	1	1	1	1	1	1	1	1	1	1	1	1	1	1	1	1	1	1	1	1	1	1	1	1	1	1	1	1	1	0	0	0	0	0	1	1	0	0	0	0	0	0	0	0	0	0	0	0	0	0	0	0	0	0	0	0	0	0	0	0	0	0	0	0	0	0	0	0	0	0	0	0	0	0	0	0	0	0	0	0	0	0
32022.283	ST50	91	1	1	1	1	1	1	1	1	1	1	1	1	1	1	1	1	1	1	1	1	1	1	1	1	1	1	1	1	1	1	1	1	1	1	1	1	1	1	1	1	1	1	1	1	1	1	1	1	1	1	1	1	1	1	1	1	1	1	1	1	1	1	1	1	1	1	1	1	1	1	1	1	1	1	1	1	1	1	1	1	1	1	1	1	1	1	1	0	0	0	0	0	1	1	0	0	0	0	0	0	0	0	0	0	0	0	0	0	0	0	0	0	0	0	0	0	0	0	0	0	0	0	0	0	0	0	0	0	0	0	0	0	0	0	0	0	0	0	0	0
32022.286	ST50	91	1	1	1	1	1	1	1	1	1	1	1	1	1	1	1	1	1	1	1	1	1	1	1	1	1	1	1	1	1	1	1	1	1	1	1	1	1	1	1	1	1	1	1	1	1	1	1	1	1	1	1	1	1	1	1	1	1	1	1	1	1	1	1	1	1	1	1	1	1	1	1	1	1	1	1	1	1	1	1	1	1	1	1	1	1	1	1	0	0	0	0	0	1	1	0	1	0	0	0	0	0	0	0	0	0	0	0	0	0	0	0	0	0	0	0	0	0	0	0	0	0	0	0	0	0	0	0	0	0	0	0	0	0	0	0	0	0	0	0	0
197.5026	ST50	92	1	1	1	1	1	1	1	1	1	1	1	1	1	1	1	1	1	1	1	1	1	1	1	1	1	1	1	1	1	1	1	1	1	1	1	1	1	1	1	1	1	1	1	1	1	1	1	1	1	1	1	1	1	1	1	1	1	1	1	1	1	1	1	1	1	1	1	1	1	1	1	1	1	1	1	1	1	1	1	1	1	1	1	1	1	1	1	0	0	0	0	0	0	1	0	1	0	0	0	0	0	0	0	0	0	0	0	0	0	0	0	0	0	0	0	0	0	0	0	0	0	0	0	0	0	0	0	0	0	0	0	0	0	0	0	0	0	0	0	0
197.5072	ST50	91	1	1	1	1	1	1	1	1	1	1	1	1	1	1	1	1	1	1	1	1	1	1	1	1	1	1	1	1	1	1	1	1	1	1	1	1	1	1	1	1	1	1	1	1	1	1	1	1	1	1	1	1	1	1	1	1	1	1	1	1	1	1	1	1	1	1	1	1	1	1	1	1	1	1	1	1	1	1	1	1	1	1	1	1	1	1	1	0	0	0	0	0	0	0	0	1	0	0	0	0	0	0	0	0	0	0	0	0	0	0	0	0	0	0	0	0	0	0	0	0	0	0	0	0	0	0	0	0	0	0	0	0	0	0	0	0	0	0	0	0
197.5184	ST50	90	1	1	1	1	1	1	1	1	1	1	1	1	1	1	1	1	1	1	1	1	1	1	1	1	1	1	1	1	1	1	1	1	1	1	1	1	1	1	1	1	1	1	1	1	1	1	1	1	1	1	1	1	1	1	1	1	1	1	1	1	1	1	1	1	1	1	1	1	1	1	1	1	1	1	1	1	1	1	1	1	1	1	1	1	1	1	1	0	0	0	0	0	0	0	0	1	0	0	0	0	0	0	0	0	0	0	0	0	0	0	0	0	0	0	0	0	0	0	0	0	0	0	0	0	0	0	0	0	0	0	0	0	0	0	0	0	0	0	0	0
889255.3	ST50	89	1	1	1	1	1	1	1	1	1	1	1	1	1	1	1	1	1	1	1	1	1	1	1	1	1	1	1	1	1	1	1	1	1	1	1	1	1	1	1	1	1	1	1	1	1	1	1	1	1	1	1	1	1	1	1	1	1	1	1	1	1	1	1	1	1	1	1	1	1	1	1	1	1	1	1	1	1	1	1	1	1	1	1	1	1	0	1	0	0	0	0	0	0	0	0	1	0	0	0	0	0	0	0	0	0	0	0	0	0	0	0	0	0	0	0	0	0	0	0	0	0	0	0	0	0	0	0	0	0	0	0	0	0	0	0	0	0	0	0	0
197.5228	ST50	89	1	1	1	1	1	1	1	1	1	1	1	1	1	1	1	1	1	1	1	1	1	1	1	1	1	1	1	1	1	1	1	1	1	1	1	1	1	1	1	1	1	1	1	1	1	1	1	1	1	1	1	1	1	1	1	1	1	1	1	1	1	1	1	1	1	1	1	1	1	1	1	1	1	1	1	1	1	1	1	1	1	1	1	1	1	1	0	0	0	0	0	0	0	0	0	1	0	0	0	0	0	0	0	0	0	0	0	0	0	0	0	0	0	0	0	0	0	0	0	0	0	0	0	0	0	0	0	0	0	0	0	0	0	0	0	0	0	0	0	0
1316917.3	ST51	88	1	1	1	1	1	1	1	1	1	1	1	1	1	1	1	1	1	1	1	1	1	1	1	1	1	1	1	1	1	1	1	1	1	1	1	1	1	1	1	1	1	1	1	1	1	1	1	1	1	1	1	1	1	1	1	1	1	1	1	1	1	1	1	1	1	1	1	1	1	1	1	1	1	1	1	1	1	1	1	1	1	1	1	1	1	0	0	0	0	0	0	0	0	0	1	0	0	0	0	0	0	0	0	0	0	0	0	0	0	0	0	0	0	0	0	0	0	0	0	0	0	0	0	0	0	0	0	0	0	0	0	0	0	0	0	0	0	0	0	0
1321759.3	ST51	88	1	1	1	1	1	1	1	1	1	1	1	1	1	1	1	1	1	1	1	1	1	1	1	1	1	1	1	1	1	1	1	1	1	1	1	1	1	1	1	1	1	1	1	1	1	1	1	1	1	1	1	1	1	1	1	1	1	1	1	1	1	1	1	1	1	1	1	1	1	1	1	1	1	1	1	1	1	1	1	1	1	1	1	1	1	0	0	0	0	0	0	0	0	0	1	0	0	0	0	0	0	0	0	0	0	0	0	0	0	0	0	0	0	0	0	0	0	0	0	0	0	0	0	0	0	0	0	0	0	0	0	0	0	0	0	0	0	0	0	0
197.11671	ST51	88	1	1	1	1	1	1	1	1	1	1	1	1	1	1	1	1	1	1	1	1	1	1	1	1	1	1	1	1	1	1	1	1	1	1	1	1	1	1	1	1	1	1	1	1	1	1	1	1	1	1	1	1	1	1	1	1	1	1	1	1	1	1	1	1	1	1	1	1	1	1	1	1	1	1	1	1	1	1	1	1	1	1	1	1	1	0	0	0	0	0	0	0	0	0	1	0	0	0	0	0	0	0	0	0	0	0	0	0	0	0	0	0	0	0	0	0	0	0	0	0	0	0	0	0	0	0	0	0	0	0	0	0	0	0	0	0	0	0	0	0
197.4827	ST51	88	1	1	1	1	1	1	1	1	1	1	1	1	1	1	1	1	1	1	1	1	1	1	1	1	1	1	1	1	1	1	1	1	1	1	1	1	1	1	1	1	1	1	1	1	1	1	1	1	1	1	1	1	1	1	1	1	1	1	1	1	1	1	1	1	1	1	1	1	1	1	1	1	1	1	1	1	1	1	1	1	1	1	1	1	1	0	0	0	0	0	0	0	0	0	1	0	0	0	0	0	0	0	0	0	0	0	0	0	0	0	0	0	0	0	0	0	0	0	0	0	0	0	0	0	0	0	0	0	0	0	0	0	0	0	0	0	0	0	0	0
197.4864	ST51	88	1	1	1	1	1	1	1	1	1	1	1	1	1	1	1	1	1	1	1	1	1	1	1	1	1	1	1	1	1	1	1	1	1	1	1	1	1	1	1	1	1	1	1	1	1	1	1	1	1	1	1	1	1	1	1	1	1	1	1	1	1	1	1	1	1	1	1	1	1	1	1	1	1	1	1	1	1	1	1	1	1	1	1	1	1	0	0	0	0	0	0	0	0	0	1	0	0	0	0	0	0	0	0	0	0	0	0	0	0	0	0	0	0	0	0	0	0	0	0	0	0	0	0	0	0	0	0	0	0	0	0	0	0	0	0	0	0	0	0	0
197.4867	ST51	88	1	1	1	1	1	1	1	1	1	1	1	1	1	1	1	1	1	1	1	1	1	1	1	1	1	1	1	1	1	1	1	1	1	1	1	1	1	1	1	1	1	1	1	1	1	1	1	1	1	1	1	1	1	1	1	1	1	1	1	1	1	1	1	1	1	1	1	1	1	1	1	1	1	1	1	1	1	1	1	1	1	1	1	1	1	0	0	0	0	0	0	0	0	0	1	0	0	0	0	0	0	0	0	0	0	0	0	0	0	0	0	0	0	0	0	0	0	0	0	0	0	0	0	0	0	0	0	0	0	0	0	0	0	0	0	0	0	0	0	0
197.4870	ST51	88	1	1	1	1	1	1	1	1	1	1	1	1	1	1	1	1	1	1	1	1	1	1	1	1	1	1	1	1	1	1	1	1	1	1	1	1	1	1	1	1	1	1	1	1	1	1	1	1	1	1	1	1	1	1	1	1	1	1	1	1	1	1	1	1	1	1	1	1	1	1	1	1	1	1	1	1	1	1	1	1	1	1	1	1	1	0	0	0	0	0	0	0	0	0	1	0	0	0	0	0	0	0	0	0	0	0	0	0	0	0	0	0	0	0	0	0	0	0	0	0	0	0	0	0	0	0	0	0	0	0	0	0	0	0	0	0	0	0	0	0
197.4975	ST51	88	1	1	1	1	1	1	1	1	1	1	1	1	1	1	1	1	1	1	1	1	1	1	1	1	1	1	1	1	1	1	1	1	1	1	1	1	1	1	1	1	1	1	1	1	1	1	1	1	1	1	1	1	1	1	1	1	1	1	1	1	1	1	1	1	1	1	1	1	1	1	1	1	1	1	1	1	1	1	1	1	1	1	1	1	1	0	0	0	0	0	0	0	0	0	1	0	0	0	0	0	0	0	0	0	0	0	0	0	0	0	0	0	0	0	0	0	0	0	0	0	0	0	0	0	0	0	0	0	0	0	0	0	0	0	0	0	0	0	0	0
197.5010	ST51	88	1	1	1	1	1	1	1	1	1	1	1	1	1	1	1	1	1	1	1	1	1	1	1	1	1	1	1	1	1	1	1	1	1	1	1	1	1	1	1	1	1	1	1	1	1	1	1	1	1	1	1	1	1	1	1	1	1	1	1	1	1	1	1	1	1	1	1	1	1	1	1	1	1	1	1	1	1	1	1	1	1	1	1	1	1	0	0	0	0	0	0	0	0	0	1	0	0	0	0	0	0	0	0	0	0	0	0	0	0	0	0	0	0	0	0	0	0	0	0	0	0	0	0	0	0	0	0	0	0	0	0	0	0	0	0	0	0	0	0	0
197.5137	ST51	88	1	1	1	1	1	1	1	1	1	1	1	1	1	1	1	1	1	1	1	1	1	1	1	1	1	1	1	1	1	1	1	1	1	1	1	1	1	1	1	1	1	1	1	1	1	1	1	1	1	1	1	1	1	1	1	1	1	1	1	1	1	1	1	1	1	1	1	1	1	1	1	1	1	1	1	1	1	1	1	1	1	1	1	1	1	0	0	0	0	0	0	0	0	0	1	0	0	0	0	0	0	0	0	0	0	0	0	0	0	0	0	0	0	0	0	0	0	0	0	0	0	0	0	0	0	0	0	0	0	0	0	0	0	0	0	0	0	0	0	0
197.5185	ST51	88	1	1	1	1	1	1	1	1	1	1	1	1	1	1	1	1	1	1	1	1	1	1	1	1	1	1	1	1	1	1	1	1	1	1	1	1	1	1	1	1	1	1	1	1	1	1	1	1	1	1	1	1	1	1	1	1	1	1	1	1	1	1	1	1	1	1	1	1	1	1	1	1	1	1	1	1	1	1	1	1	1	1	1	1	1	0	0	0	0	0	0	0	0	0	1	0	0	0	0	0	0	0	0	0	0	0	0	0	0	0	0	0	0	0	0	0	0	0	0	0	0	0	0	0	0	0	0	0	0	0	0	0	0	0	0	0	0	0	0	0
32022.608	ST51	88	1	1	1	1	1	1	1	1	1	1	1	1	1	1	1	1	1	1	1	1	1	1	1	1	1	1	1	1	1	1	1	1	1	1	1	1	1	1	1	1	1	1	1	1	1	1	1	1	1	1	1	1	1	1	1	1	1	1	1	1	1	1	1	1	1	1	1	1	1	1	1	1	1	1	1	1	1	1	1	1	1	1	1	1	1	0	0	0	0	0	0	0	0	0	1	0	0	0	0	0	0	0	0	0	0	0	0	0	0	0	0	0	0	0	0	0	0	0	0	0	0	0	0	0	0	0	0	0	0	0	0	0	0	0	0	0	0	0	0	0
32022.609	ST51	88	1	1	1	1	1	1	1	1	1	1	1	1	1	1	1	1	1	1	1	1	1	1	1	1	1	1	1	1	1	1	1	1	1	1	1	1	1	1	1	1	1	1	1	1	1	1	1	1	1	1	1	1	1	1	1	1	1	1	1	1	1	1	1	1	1	1	1	1	1	1	1	1	1	1	1	1	1	1	1	1	1	1	1	1	1	0	0	0	0	0	0	0	0	0	1	0	0	0	0	0	0	0	0	0	0	0	0	0	0	0	0	0	0	0	0	0	0	0	0	0	0	0	0	0	0	0	0	0	0	0	0	0	0	0	0	0	0	0	0	0
32022.610	ST51	88	1	1	1	1	1	1	1	1	1	1	1	1	1	1	1	1	1	1	1	1	1	1	1	1	1	1	1	1	1	1	1	1	1	1	1	1	1	1	1	1	1	1	1	1	1	1	1	1	1	1	1	1	1	1	1	1	1	1	1	1	1	1	1	1	1	1	1	1	1	1	1	1	1	1	1	1	1	1	1	1	1	1	1	1	1	0	0	0	0	0	0	0	0	0	1	0	0	0	0	0	0	0	0	0	0	0	0	0	0	0	0	0	0	0	0	0	0	0	0	0	0	0	0	0	0	0	0	0	0	0	0	0	0	0	0	0	0	0	0	0
197.5088	ST51	89	1	1	1	1	1	1	1	1	1	1	1	1	1	1	1	1	1	1	1	1	1	1	1	1	1	1	1	1	1	1	1	1	1	1	1	1	1	1	1	1	1	1	1	1	1	1	1	1	1	1	1	1	1	1	1	1	1	1	1	1	1	1	1	1	1	1	1	1	1	1	1	1	1	1	1	1	1	1	1	1	1	1	1	1	1	0	0	0	0	0	0	0	0	0	1	1	0	0	0	0	0	0	0	0	0	0	0	0	0	0	0	0	0	0	0	0	0	0	0	0	0	0	0	0	0	0	0	0	0	0	0	0	0	0	0	0	0	0	0	0
197.5043	ST51	89	1	1	1	1	1	1	1	1	1	1	1	1	1	1	1	1	1	1	1	1	1	1	1	1	1	1	1	1	1	1	1	1	1	1	1	1	1	1	1	1	1	1	1	1	1	1	1	1	1	1	1	1	1	1	1	1	1	1	1	1	1	1	1	1	1	1	1	1	1	1	1	1	1	1	1	1	1	1	1	1	1	1	1	1	1	1	0	0	0	0	0	0	0	0	1	0	0	0	0	0	0	0	0	0	0	0	0	0	0	0	0	0	0	0	0	0	0	0	0	0	0	0	0	0	0	0	0	0	0	0	0	0	0	0	1	0	0	0	0	0
197.5069	ST51	89	1	1	1	1	1	1	1	1	1	1	1	1	1	1	1	1	1	1	1	1	1	1	1	1	1	1	1	1	1	1	1	1	1	1	1	1	1	1	1	1	1	1	1	1	1	1	1	1	1	1	1	1	1	1	1	1	1	1	1	1	1	1	1	1	1	1	1	1	1	1	1	1	1	1	1	1	1	1	1	1	1	1	1	1	1	0	0	0	0	0	0	0	0	0	1	0	0	0	0	0	0	0	0	0	0	0	0	0	0	0	0	0	0	0	0	0	0	0	0	0	0	0	0	0	0	0	0	0	0	0	0	0	0	0	0	0	0	0	0	0
197.5167	ST51	87	1	1	1	1	1	1	1	1	1	1	1	1	1	1	1	1	1	1	1	1	1	1	1	1	1	1	1	1	1	1	1	1	1	1	1	1	1	1	1	1	1	1	1	1	1	1	1	1	1	1	1	1	1	1	1	1	1	1	1	1	1	1	1	1	1	1	1	1	1	1	1	1	1	1	1	1	1	1	1	1	1	1	1	0	1	0	0	0	0	0	0	0	0	0	1	0	0	0	0	0	0	0	0	0	0	0	0	0	0	0	0	0	0	0	0	0	0	0	0	0	0	0	0	0	0	0	0	0	0	0	0	0	0	0	0	0	0	0	0	0
197.7745	ST51	90	1	1	1	1	1	1	1	1	1	1	1	1	1	1	1	1	1	1	1	1	1	1	1	1	1	1	1	1	1	1	1	1	1	1	1	1	1	1	1	1	1	1	1	1	1	1	1	1	1	1	1	1	1	1	1	1	1	1	1	1	1	1	1	1	1	1	1	1	1	1	1	1	1	1	1	1	1	1	1	1	1	1	1	1	1	0	0	0	0	0	0	0	1	1	1	0	0	0	0	0	0	0	0	0	0	0	0	0	0	0	0	0	0	0	0	0	0	0	0	0	0	0	0	0	0	0	0	0	0	0	0	0	0	0	0	0	0	0	0	0
197.7920	ST51	90	1	1	1	1	1	1	1	1	1	1	1	1	1	1	1	1	1	1	1	1	1	1	1	1	1	1	1	1	1	1	1	1	1	1	1	1	1	1	1	1	1	1	1	1	1	1	1	1	1	1	1	1	1	1	1	1	1	1	1	1	1	1	1	1	1	1	1	1	1	1	1	1	1	1	1	1	1	1	1	1	1	1	1	1	1	0	0	0	0	0	0	0	1	1	1	0	0	0	0	0	0	0	0	0	0	0	0	0	0	0	0	0	0	0	0	0	0	0	0	0	0	0	0	0	0	0	0	0	0	0	0	0	0	0	0	0	0	0	0	0
197.7921	ST51	90	1	1	1	1	1	1	1	1	1	1	1	1	1	1	1	1	1	1	1	1	1	1	1	1	1	1	1	1	1	1	1	1	1	1	1	1	1	1	1	1	1	1	1	1	1	1	1	1	1	1	1	1	1	1	1	1	1	1	1	1	1	1	1	1	1	1	1	1	1	1	1	1	1	1	1	1	1	1	1	1	1	1	1	1	1	0	0	0	0	0	0	0	1	1	1	0	0	0	0	0	0	0	0	0	0	0	0	0	0	0	0	0	0	0	0	0	0	0	0	0	0	0	0	0	0	0	0	0	0	0	0	0	0	0	0	0	0	0	0	0
197.4956	ST51	89	1	1	1	1	1	1	1	1	1	1	1	1	1	1	1	1	1	1	1	1	1	1	1	1	1	1	1	1	1	1	1	1	1	1	1	1	1	1	1	1	1	1	1	1	1	1	1	1	1	1	1	1	1	1	1	1	1	1	1	1	1	1	1	1	1	1	1	1	1	1	1	1	1	1	1	1	1	1	1	1	1	1	1	1	1	0	0	0	0	0	0	0	0	1	1	0	0	0	0	0	0	0	0	0	0	0	0	0	0	0	0	0	0	0	0	0	0	0	0	0	0	0	0	0	0	0	0	0	0	0	0	0	0	0	0	0	0	0	0	0
197.4994	ST51	89	1	1	1	1	1	1	1	1	1	1	1	1	1	1	1	1	1	1	1	1	1	1	1	1	1	1	1	1	1	1	1	1	1	1	1	1	1	1	1	1	1	1	1	1	1	1	1	1	1	1	1	1	1	1	1	1	1	1	1	1	1	1	1	1	1	1	1	1	1	1	1	1	1	1	1	1	1	1	1	1	1	1	1	1	1	0	0	0	0	0	0	0	0	1	1	0	0	0	0	0	0	0	0	0	0	0	0	0	0	0	0	0	0	0	0	0	0	0	0	0	0	0	0	0	0	0	0	0	0	0	0	0	0	0	0	0	0	0	0	0
KF070	ST51	89	1	1	1	1	1	1	1	1	1	1	1	1	1	1	1	1	1	1	1	1	1	1	1	1	1	1	1	1	1	1	1	1	1	1	1	1	1	1	1	1	1	1	1	1	1	1	1	1	1	1	1	1	1	1	1	1	1	1	1	1	1	1	1	1	1	1	1	1	1	1	1	1	1	1	1	1	1	1	1	1	1	1	1	1	1	0	0	0	0	0	0	0	0	0	1	0	0	0	0	0	0	0	0	0	0	0	0	0	0	0	0	0	0	0	0	0	0	0	0	0	0	0	0	0	0	0	0	0	0	0	0	0	0	0	0	0	0	0	0	0
197.5174	ST51	86	1	1	1	1	0	0	1	1	1	1	1	1	1	1	1	1	1	1	1	1	1	1	1	1	1	1	1	1	1	1	1	1	1	1	1	1	1	1	1	1	1	1	1	1	1	1	1	1	1	1	1	1	1	1	1	1	1	1	1	1	1	1	1	1	1	1	1	1	1	1	1	1	1	1	1	1	1	1	1	1	1	1	1	1	1	0	0	0	0	0	0	0	0	0	1	0	0	0	0	0	0	0	0	0	0	0	0	0	0	0	0	0	0	0	0	0	0	0	0	0	0	0	0	0	0	0	0	0	0	0	0	0	0	0	0	0	0	0	0	0
1440029.3	ST50	94	1	1	1	1	1	1	1	1	1	1	1	1	1	1	1	1	1	1	1	1	1	1	1	1	1	1	1	1	1	1	1	1	1	1	1	1	1	1	1	1	1	1	1	1	1	1	1	1	1	1	1	1	1	1	1	1	1	1	1	1	1	1	1	1	1	1	1	1	1	1	1	1	1	1	1	1	1	1	1	1	1	1	1	1	1	0	1	1	1	1	1	1	1	0	0	0	0	0	0	0	0	0	0	0	0	0	0	0	0	0	0	0	0	0	0	0	0	0	0	0	0	0	0	0	0	0	0	0	0	0	0	0	0	0	0	0	0	0	0	0
197.11580	ST50	95	1	1	1	1	1	1	1	1	1	1	1	1	1	1	1	1	1	1	1	1	1	1	1	1	1	1	1	1	1	1	1	1	1	1	1	1	1	1	1	1	1	1	1	1	1	1	1	1	1	1	1	1	1	1	1	1	1	1	1	1	1	1	1	1	1	1	1	1	1	1	1	1	1	1	1	1	1	1	1	1	1	1	1	1	1	0	1	1	1	1	1	1	0	0	0	0	0	0	0	0	0	0	0	0	0	0	0	0	0	0	0	0	0	0	0	0	0	0	0	0	0	0	0	0	0	0	0	0	0	0	0	0	0	0	0	0	0	0	0	0
889228.3	ST50	92	1	1	1	1	1	1	1	1	1	1	1	1	1	1	1	1	1	1	1	1	1	1	1	1	1	1	1	1	1	1	1	1	1	1	1	1	1	1	1	1	1	1	1	1	1	1	1	1	1	1	1	1	1	1	1	1	1	1	1	1	1	1	1	1	1	1	1	1	1	1	1	1	1	1	1	1	1	1	1	1	1	1	1	1	1	0	1	1	1	1	1	0	0	0	0	0	0	0	0	0	0	0	0	0	0	0	0	0	0	0	0	0	0	0	0	0	0	0	0	0	0	0	0	0	0	0	0	0	0	0	0	0	0	0	0	0	0	0	0	0
197.4950	ST50	96	1	1	1	1	1	1	1	1	1	1	1	1	1	1	1	1	1	1	1	1	1	1	1	1	1	1	1	1	1	1	1	1	1	1	1	1	1	1	1	1	1	1	1	1	1	1	1	1	1	1	1	1	1	1	1	1	1	1	1	1	1	1	1	1	1	1	1	1	1	1	1	1	1	1	1	1	1	1	1	1	1	1	1	1	1	1	1	1	1	1	1	1	0	0	0	1	0	0	0	0	0	0	0	0	0	0	0	0	0	0	0	0	0	1	0	0	0	0	0	0	0	0	0	0	0	0	0	0	0	0	0	0	0	0	0	0	0	0	0	0
197.5099	ST50	96	1	1	1	1	1	1	1	1	1	1	1	1	1	1	1	1	1	1	1	1	1	1	1	1	1	1	1	1	1	1	1	1	1	1	1	1	1	1	1	1	1	1	1	1	1	1	1	1	1	1	1	1	1	1	1	1	1	1	1	1	1	1	1	1	1	1	1	1	1	1	1	1	1	1	1	1	1	1	1	1	1	1	1	1	1	1	1	1	1	1	1	1	0	0	0	1	0	0	0	0	0	0	0	0	0	0	0	0	0	0	0	0	0	1	0	0	0	0	0	0	0	0	0	0	0	0	0	0	0	0	0	0	0	0	0	0	0	0	0	0
KF017	ST50	95	1	1	1	1	1	1	1	1	1	1	1	1	1	1	1	1	1	1	1	1	1	1	1	1	1	1	1	1	1	1	1	1	1	1	1	1	1	1	1	1	1	1	1	1	1	1	1	1	1	1	1	1	1	1	1	1	1	1	1	1	1	1	1	1	1	1	1	1	1	1	1	1	1	1	1	1	1	1	1	1	1	1	1	1	1	0	1	1	1	1	1	1	0	0	0	1	0	0	0	0	0	0	0	0	0	0	0	0	0	0	0	0	0	0	0	0	0	0	0	0	0	0	0	0	0	0	0	0	0	0	0	0	0	0	0	0	0	0	0	0
889250.3	ST50	94	1	1	1	1	1	1	1	1	1	1	1	1	1	1	1	1	1	1	1	1	1	1	1	1	1	1	1	1	1	1	1	1	1	1	1	1	1	1	1	1	1	1	1	1	1	1	1	1	1	1	1	1	1	1	1	1	1	1	1	1	1	1	1	1	1	1	1	1	1	1	1	1	1	1	1	1	1	1	1	1	1	1	1	1	1	1	1	1	1	1	1	1	0	1	0	0	0	0	0	0	0	0	0	0	0	0	0	0	0	0	0	0	0	0	0	0	0	0	0	0	0	0	0	0	0	0	0	0	0	0	0	0	0	0	0	0	0	0	0	0
197.5004	ST50	95	1	1	1	1	1	1	1	1	1	1	1	1	1	1	1	1	1	1	1	1	1	1	1	1	1	1	1	1	1	1	1	1	1	1	1	1	1	1	1	1	1	1	1	1	1	1	1	1	1	1	1	1	1	1	1	1	1	1	1	1	1	1	1	1	1	1	1	1	1	1	1	1	1	1	1	1	1	1	1	1	1	1	1	1	1	1	1	1	1	1	1	1	0	1	0	0	0	0	0	0	0	0	0	0	0	0	0	0	0	0	0	0	0	0	0	0	0	0	0	0	0	0	0	0	0	0	0	0	0	0	0	0	0	0	0	0	0	0	0	0
197.6758	ST50	95	1	1	1	1	1	1	1	1	1	1	1	1	1	1	1	1	1	1	1	1	1	1	1	1	1	1	1	1	1	1	1	1	1	1	1	1	1	1	1	1	1	1	1	1	1	1	1	1	1	1	1	1	1	1	1	1	1	1	1	1	1	1	1	1	1	1	1	1	1	1	1	1	1	1	1	1	1	1	1	1	1	1	1	1	1	1	1	1	1	1	1	1	0	0	0	0	0	0	0	0	0	0	0	0	0	0	0	0	0	0	0	0	0	0	0	0	0	0	0	0	0	0	0	0	0	0	0	0	0	0	0	0	0	0	0	0	0	0	0	0
197.4814	ST50	94	1	1	1	1	1	1	1	1	1	1	1	1	1	1	1	1	1	1	1	1	1	1	1	1	1	1	1	1	1	1	1	1	1	1	1	1	1	1	1	1	1	1	1	1	1	1	1	1	1	1	1	1	1	1	1	1	1	1	1	1	1	1	1	1	1	1	1	1	1	1	1	1	1	1	1	1	1	1	1	1	1	1	1	1	1	1	1	1	1	1	1	1	0	0	0	0	0	0	0	0	0	0	0	0	0	0	0	0	0	0	0	0	0	0	0	0	0	0	0	0	0	0	0	0	0	0	0	0	0	0	0	0	0	0	0	0	0	0	0	0
197.4816	ST50	94	1	1	1	1	1	1	1	1	1	1	1	1	1	1	1	1	1	1	1	1	1	1	1	1	1	1	1	1	1	1	1	1	1	1	1	1	1	1	1	1	1	1	1	1	1	1	1	1	1	1	1	1	1	1	1	1	1	1	1	1	1	1	1	1	1	1	1	1	1	1	1	1	1	1	1	1	1	1	1	1	1	1	1	1	1	1	1	1	1	1	1	1	0	0	0	0	0	0	0	0	0	0	0	0	0	0	0	0	0	0	0	0	0	0	0	0	0	0	0	0	0	0	0	0	0	0	0	0	0	0	0	0	0	0	0	0	0	0	0	0
197.4824	ST50	94	1	1	1	1	1	1	1	1	1	1	1	1	1	1	1	1	1	1	1	1	1	1	1	1	1	1	1	1	1	1	1	1	1	1	1	1	1	1	1	1	1	1	1	1	1	1	1	1	1	1	1	1	1	1	1	1	1	1	1	1	1	1	1	1	1	1	1	1	1	1	1	1	1	1	1	1	1	1	1	1	1	1	1	1	1	1	1	1	1	1	1	1	0	0	0	0	0	0	0	0	0	0	0	0	0	0	0	0	0	0	0	0	0	0	0	0	0	0	0	0	0	0	0	0	0	0	0	0	0	0	0	0	0	0	0	0	0	0	0	0
197.4871	ST50	94	1	1	1	1	1	1	1	1	1	1	1	1	1	1	1	1	1	1	1	1	1	1	1	1	1	1	1	1	1	1	1	1	1	1	1	1	1	1	1	1	1	1	1	1	1	1	1	1	1	1	1	1	1	1	1	1	1	1	1	1	1	1	1	1	1	1	1	1	1	1	1	1	1	1	1	1	1	1	1	1	1	1	1	1	1	1	1	1	1	1	1	1	0	0	0	0	0	0	0	0	0	0	0	0	0	0	0	0	0	0	0	0	0	0	0	0	0	0	0	0	0	0	0	0	0	0	0	0	0	0	0	0	0	0	0	0	0	0	0	0
197.4901	ST50	94	1	1	1	1	1	1	1	1	1	1	1	1	1	1	1	1	1	1	1	1	1	1	1	1	1	1	1	1	1	1	1	1	1	1	1	1	1	1	1	1	1	1	1	1	1	1	1	1	1	1	1	1	1	1	1	1	1	1	1	1	1	1	1	1	1	1	1	1	1	1	1	1	1	1	1	1	1	1	1	1	1	1	1	1	1	1	1	1	1	1	1	1	0	0	0	0	0	0	0	0	0	0	0	0	0	0	0	0	0	0	0	0	0	0	0	0	0	0	0	0	0	0	0	0	0	0	0	0	0	0	0	0	0	0	0	0	0	0	0	0
197.4910	ST50	94	1	1	1	1	1	1	1	1	1	1	1	1	1	1	1	1	1	1	1	1	1	1	1	1	1	1	1	1	1	1	1	1	1	1	1	1	1	1	1	1	1	1	1	1	1	1	1	1	1	1	1	1	1	1	1	1	1	1	1	1	1	1	1	1	1	1	1	1	1	1	1	1	1	1	1	1	1	1	1	1	1	1	1	1	1	1	1	1	1	1	1	1	0	0	0	0	0	0	0	0	0	0	0	0	0	0	0	0	0	0	0	0	0	0	0	0	0	0	0	0	0	0	0	0	0	0	0	0	0	0	0	0	0	0	0	0	0	0	0	0
197.4925	ST50	94	1	1	1	1	1	1	1	1	1	1	1	1	1	1	1	1	1	1	1	1	1	1	1	1	1	1	1	1	1	1	1	1	1	1	1	1	1	1	1	1	1	1	1	1	1	1	1	1	1	1	1	1	1	1	1	1	1	1	1	1	1	1	1	1	1	1	1	1	1	1	1	1	1	1	1	1	1	1	1	1	1	1	1	1	1	1	1	1	1	1	1	1	0	0	0	0	0	0	0	0	0	0	0	0	0	0	0	0	0	0	0	0	0	0	0	0	0	0	0	0	0	0	0	0	0	0	0	0	0	0	0	0	0	0	0	0	0	0	0	0
197.5020	ST50	94	1	1	1	1	1	1	1	1	1	1	1	1	1	1	1	1	1	1	1	1	1	1	1	1	1	1	1	1	1	1	1	1	1	1	1	1	1	1	1	1	1	1	1	1	1	1	1	1	1	1	1	1	1	1	1	1	1	1	1	1	1	1	1	1	1	1	1	1	1	1	1	1	1	1	1	1	1	1	1	1	1	1	1	1	1	1	1	1	1	1	1	1	0	0	0	0	0	0	0	0	0	0	0	0	0	0	0	0	0	0	0	0	0	0	0	0	0	0	0	0	0	0	0	0	0	0	0	0	0	0	0	0	0	0	0	0	0	0	0	0
197.5038	ST50	94	1	1	1	1	1	1	1	1	1	1	1	1	1	1	1	1	1	1	1	1	1	1	1	1	1	1	1	1	1	1	1	1	1	1	1	1	1	1	1	1	1	1	1	1	1	1	1	1	1	1	1	1	1	1	1	1	1	1	1	1	1	1	1	1	1	1	1	1	1	1	1	1	1	1	1	1	1	1	1	1	1	1	1	1	1	1	1	1	1	1	1	1	0	0	0	0	0	0	0	0	0	0	0	0	0	0	0	0	0	0	0	0	0	0	0	0	0	0	0	0	0	0	0	0	0	0	0	0	0	0	0	0	0	0	0	0	0	0	0	0
197.5092	ST50	94	1	1	1	1	1	1	1	1	1	1	1	1	1	1	1	1	1	1	1	1	1	1	1	1	1	1	1	1	1	1	1	1	1	1	1	1	1	1	1	1	1	1	1	1	1	1	1	1	1	1	1	1	1	1	1	1	1	1	1	1	1	1	1	1	1	1	1	1	1	1	1	1	1	1	1	1	1	1	1	1	1	1	1	1	1	1	1	1	1	1	1	1	0	0	0	0	0	0	0	0	0	0	0	0	0	0	0	0	0	0	0	0	0	0	0	0	0	0	0	0	0	0	0	0	0	0	0	0	0	0	0	0	0	0	0	0	0	0	0	0
197.5113	ST50	94	1	1	1	1	1	1	1	1	1	1	1	1	1	1	1	1	1	1	1	1	1	1	1	1	1	1	1	1	1	1	1	1	1	1	1	1	1	1	1	1	1	1	1	1	1	1	1	1	1	1	1	1	1	1	1	1	1	1	1	1	1	1	1	1	1	1	1	1	1	1	1	1	1	1	1	1	1	1	1	1	1	1	1	1	1	1	1	1	1	1	1	1	0	0	0	0	0	0	0	0	0	0	0	0	0	0	0	0	0	0	0	0	0	0	0	0	0	0	0	0	0	0	0	0	0	0	0	0	0	0	0	0	0	0	0	0	0	0	0	0
197.5133	ST50	94	1	1	1	1	1	1	1	1	1	1	1	1	1	1	1	1	1	1	1	1	1	1	1	1	1	1	1	1	1	1	1	1	1	1	1	1	1	1	1	1	1	1	1	1	1	1	1	1	1	1	1	1	1	1	1	1	1	1	1	1	1	1	1	1	1	1	1	1	1	1	1	1	1	1	1	1	1	1	1	1	1	1	1	1	1	1	1	1	1	1	1	1	0	0	0	0	0	0	0	0	0	0	0	0	0	0	0	0	0	0	0	0	0	0	0	0	0	0	0	0	0	0	0	0	0	0	0	0	0	0	0	0	0	0	0	0	0	0	0	0
32022.634	ST50	94	1	1	1	1	1	1	1	1	1	1	1	1	1	1	1	1	1	1	1	1	1	1	1	1	1	1	1	1	1	1	1	1	1	1	1	1	1	1	1	1	1	1	1	1	1	1	1	1	1	1	1	1	1	1	1	1	1	1	1	1	1	1	1	1	1	1	1	1	1	1	1	1	1	1	1	1	1	1	1	1	1	1	1	1	1	1	1	1	1	1	1	1	0	0	0	0	0	0	0	0	0	0	0	0	0	0	0	0	0	0	0	0	0	0	0	0	0	0	0	0	0	0	0	0	0	0	0	0	0	0	0	0	0	0	0	0	0	0	0	0
197.5035	ST50	95	1	1	1	1	1	1	1	1	1	1	1	1	1	1	1	1	1	1	1	1	1	1	1	1	1	1	1	1	1	1	1	1	1	1	1	1	1	1	1	1	1	1	1	1	1	1	1	1	1	1	1	1	1	1	1	1	1	1	1	1	1	1	1	1	1	1	1	1	1	1	1	1	1	1	1	1	1	1	1	1	1	1	1	1	1	1	1	1	1	1	1	1	0	0	0	0	0	0	0	0	0	0	0	0	0	0	0	0	0	0	0	0	0	0	0	0	0	0	0	0	0	0	0	0	0	0	0	0	0	0	0	1	0	0	0	0	0	0
1440034.3	ST50	93	1	1	1	1	1	1	1	1	1	1	1	1	1	1	1	1	1	1	1	1	1	1	1	1	1	1	1	1	1	1	1	1	1	1	1	1	1	1	1	1	1	1	1	1	1	1	1	1	1	1	1	1	1	1	1	1	1	1	1	1	1	1	1	1	1	1	1	1	1	1	1	1	1	1	1	1	1	1	1	1	0	1	1	1	1	1	1	1	1	1	1	1	0	0	0	0	0	0	0	0	0	0	0	0	0	0	0	0	0	0	0	0	0	0	0	0	0	0	0	0	0	0	0	0	0	0	0	0	0	0	0	0	0	0	0	0	0	0	0	0
197.4838	ST50	95	1	1	1	1	1	1	1	1	1	1	1	1	1	1	1	1	1	1	1	1	1	1	1	1	1	1	1	1	1	1	1	1	1	1	1	1	1	1	1	1	1	1	1	1	1	1	1	1	1	1	1	1	1	1	1	1	1	1	1	1	1	1	1	1	1	1	1	1	1	1	1	1	1	1	1	1	1	1	1	1	1	1	1	1	1	1	1	1	1	1	1	1	1	0	0	0	0	0	0	0	0	0	0	0	0	0	0	0	0	0	0	0	0	0	0	0	0	0	0	0	0	0	0	0	0	0	0	0	0	0	0	0	0	0	0	0	0	0	0	0
197.5001	ST50	95	1	1	1	1	1	1	1	1	1	1	1	1	1	1	1	1	1	1	1	1	1	1	1	1	1	1	1	1	1	1	1	1	1	1	1	1	1	1	1	1	1	1	1	1	1	1	1	1	1	1	1	1	1	1	1	1	1	1	1	1	1	1	1	1	1	1	1	1	1	1	1	1	1	1	1	1	1	1	1	1	1	1	1	1	1	1	1	1	1	1	1	1	1	0	0	0	0	0	0	0	0	0	0	0	0	0	0	0	0	0	0	0	0	0	0	0	0	0	0	0	0	0	0	0	0	0	0	0	0	0	0	0	0	0	0	0	0	0	0	0
197.5076	ST50	95	1	1	1	1	1	1	1	1	1	1	1	1	1	1	1	1	1	1	1	1	1	1	1	1	1	1	1	1	1	1	1	1	1	1	1	1	1	1	1	1	1	1	1	1	1	1	1	1	1	1	1	1	1	1	1	1	1	1	1	1	1	1	1	1	1	1	1	1	1	1	1	1	1	1	1	1	1	1	1	1	1	1	1	1	1	1	1	1	1	1	1	1	1	0	0	0	0	0	0	0	0	0	0	0	0	0	0	0	0	0	0	0	0	0	0	0	0	0	0	0	0	0	0	0	0	0	0	0	0	0	0	0	0	0	0	0	0	0	0	0
197.6746	ST50	95	1	1	1	1	1	1	1	1	1	1	1	1	1	1	1	1	1	1	1	1	1	1	1	1	1	1	1	1	1	1	1	1	1	1	1	1	1	1	1	1	1	1	1	1	1	1	1	1	1	1	1	1	1	1	1	1	1	1	1	1	1	1	1	1	1	1	1	1	1	1	1	1	1	1	1	1	1	1	1	1	1	1	1	1	1	1	1	1	1	1	1	1	1	1	0	0	0	0	0	0	0	0	0	0	0	0	0	0	0	0	0	0	0	0	0	0	0	0	0	0	0	0	0	0	0	0	0	0	0	0	0	0	0	0	0	0	0	0	0	0
197.6704	ST50	96	1	1	1	1	1	1	1	1	1	1	1	1	1	1	1	1	1	1	1	1	1	1	1	1	1	1	1	1	1	1	1	1	1	1	1	1	1	1	1	1	1	1	1	1	1	1	1	1	1	1	1	1	1	1	1	1	1	1	1	1	1	1	1	1	1	1	1	1	1	1	1	1	1	1	1	1	1	1	1	1	1	1	1	1	1	1	1	1	1	1	1	1	1	1	0	0	0	0	0	0	0	0	0	0	0	0	0	0	0	0	0	0	0	0	0	0	0	0	0	0	0	0	0	0	0	0	0	0	0	0	0	0	0	0	0	0	0	0	0	0
197.4952	ST50	95	1	1	1	1	1	1	1	1	1	1	1	1	1	1	1	1	1	1	1	1	1	1	1	1	1	1	1	1	1	1	1	1	1	1	1	1	1	1	1	1	1	1	1	1	1	1	1	1	1	1	1	1	1	1	1	1	1	1	1	1	1	1	1	1	1	1	1	1	1	1	1	1	1	1	1	1	1	1	1	1	1	1	0	1	1	1	1	1	1	1	1	1	1	0	0	1	0	0	0	0	0	0	0	0	0	0	0	0	0	0	0	0	0	0	0	0	0	0	0	0	0	0	0	0	0	0	0	0	0	0	0	0	0	0	0	0	0	0	0	0
197.4983	ST50	96	1	1	1	1	1	1	1	1	1	1	1	1	1	1	1	1	1	1	1	1	1	1	1	1	1	1	1	1	1	1	1	1	1	1	1	1	1	1	1	1	1	1	1	1	1	1	1	1	1	1	1	1	1	1	1	1	1	1	1	1	1	1	1	1	1	1	1	1	1	1	1	1	1	1	1	1	1	1	1	1	1	1	1	1	1	1	1	1	1	1	1	1	1	0	0	1	0	0	0	0	0	0	0	0	0	0	0	0	0	0	0	0	0	0	0	0	0	0	0	0	0	0	0	0	0	0	0	0	0	0	0	0	0	0	0	0	0	0	0	0
197.13052	ST50	97	1	1	1	1	1	1	1	1	1	1	1	1	1	1	1	1	1	1	1	1	1	1	1	1	1	1	1	1	1	1	1	1	1	1	1	1	1	1	1	1	1	1	1	1	1	1	1	1	1	1	1	1	1	1	1	1	1	1	1	1	1	1	1	1	1	1	1	1	1	1	1	1	1	1	1	1	1	1	1	1	1	1	1	1	1	1	1	1	1	1	1	1	1	1	0	1	0	0	0	0	0	0	0	0	0	0	0	0	0	0	0	0	0	0	0	0	0	0	0	0	0	0	0	0	0	0	0	0	0	0	0	0	0	0	0	0	0	0	0	0
197.5003	ST50	98	1	1	1	1	1	1	1	1	1	1	1	1	1	1	1	1	1	1	1	1	1	1	1	1	1	1	1	1	1	1	1	1	1	1	1	1	1	1	1	1	1	1	1	1	1	1	1	1	1	1	1	1	1	1	1	1	1	1	1	1	1	1	1	1	1	1	1	1	1	1	1	1	1	1	1	1	1	1	1	1	1	1	1	1	1	1	1	1	1	1	1	1	0	0	0	1	0	0	0	0	0	0	0	0	0	0	0	0	0	0	0	0	0	1	0	0	0	0	0	0	0	0	0	0	0	0	0	0	0	0	0	0	0	0	0	0	0	0	0	0
KF042	ST50	93	1	1	1	1	1	1	1	1	1	1	1	1	1	1	1	1	1	1	1	1	1	1	1	1	1	1	1	1	1	1	1	1	1	1	1	1	1	1	1	1	1	1	1	1	1	1	1	1	1	1	1	1	1	1	1	1	1	1	1	1	1	1	1	1	1	1	1	1	1	1	1	1	1	1	1	1	1	1	0	0	1	1	1	1	1	1	1	1	1	1	1	1	1	1	0	1	0	0	0	0	0	0	0	0	0	0	0	0	0	0	0	0	0	0	0	0	0	0	0	0	0	0	0	0	0	0	0	0	0	0	0	0	0	0	0	0	0	0	0	0
32022.288	ST50	94	0	0	1	1	1	1	1	1	1	1	1	1	1	1	1	1	1	1	1	1	1	1	1	1	1	1	1	1	1	1	1	1	1	1	1	1	1	1	1	1	1	1	1	1	1	1	1	1	1	1	1	1	1	1	1	1	1	1	1	1	1	1	1	1	1	1	1	1	1	1	1	1	1	1	1	1	1	1	1	1	1	1	1	1	0	0	1	1	1	1	1	1	1	1	0	1	0	0	0	0	0	0	0	0	0	0	0	0	0	0	0	0	0	0	1	0	0	0	0	0	0	0	0	0	0	0	0	0	0	0	0	0	0	0	0	0	0	0	0	0
32022.290	ST50	94	0	0	1	1	1	1	1	1	1	1	1	1	1	1	1	1	1	1	1	1	1	1	1	1	1	1	1	1	1	1	1	1	1	1	1	1	1	1	1	1	1	1	1	1	1	1	1	1	1	1	1	1	1	1	1	1	1	1	1	1	1	1	1	1	1	1	1	1	1	1	1	1	1	1	1	1	1	1	1	1	1	1	1	1	0	0	1	1	1	1	1	1	0	0	0	1	0	0	0	0	0	0	0	0	0	0	0	0	0	0	0	0	0	0	0	0	0	0	0	0	0	0	0	0	0	0	0	0	0	0	0	0	0	0	0	0	0	0	0	0
32022.285	ST50	92	0	0	1	1	1	1	1	1	1	1	1	1	1	1	1	1	1	1	1	1	1	1	1	1	1	1	1	1	1	1	1	1	1	1	1	1	1	1	1	1	1	1	1	1	1	1	1	1	1	1	1	1	1	1	1	1	1	1	1	1	1	1	1	1	1	1	1	1	1	1	1	1	1	1	1	1	1	1	1	1	1	1	1	1	0	0	1	1	1	1	1	1	1	1	0	1	0	0	0	0	0	0	0	0	0	0	0	0	0	0	0	0	0	0	1	0	0	0	0	0	0	0	0	0	0	0	0	0	0	0	0	0	0	0	0	0	0	0	0	0
32022.279	ST50	88	0	0	1	1	1	1	1	1	1	1	1	1	1	1	1	1	1	1	1	1	1	1	1	1	1	1	1	1	1	1	1	1	1	1	1	1	1	1	1	1	1	1	1	1	1	1	1	1	1	1	1	1	1	1	1	1	1	1	1	1	1	1	1	1	1	1	1	1	1	1	1	1	1	1	1	1	1	1	1	1	1	1	1	1	0	0	1	0	0	0	0	0	1	1	0	1	0	0	0	0	0	0	0	0	0	0	0	0	0	0	0	0	0	0	0	0	0	0	0	0	0	0	0	0	0	0	0	0	0	0	0	0	0	0	0	0	0	0	0	0
32022.280	ST50	88	0	0	1	1	1	1	1	1	1	1	1	1	1	1	1	1	1	1	1	1	1	1	1	1	1	1	1	1	1	1	1	1	1	1	1	1	1	1	1	1	1	1	1	1	1	1	1	1	1	1	1	1	1	1	1	1	1	1	1	1	1	1	1	1	1	1	1	1	1	1	1	1	1	1	1	1	1	1	1	1	1	1	1	1	0	0	1	0	0	0	0	0	1	1	0	1	0	0	0	0	0	0	0	0	0	0	0	0	0	0	0	0	0	0	0	0	0	0	0	0	0	0	0	0	0	0	0	0	0	0	0	0	0	0	0	0	0	0	0	0
32022.282	ST50	88	0	0	1	1	1	1	1	1	1	1	1	1	1	1	1	1	1	1	1	1	1	1	1	1	1	1	1	1	1	1	1	1	1	1	1	1	1	1	1	1	1	1	1	1	1	1	1	1	1	1	1	1	1	1	1	1	1	1	1	1	1	1	1	1	1	1	1	1	1	1	1	1	1	1	1	1	1	1	1	1	1	1	1	1	0	0	1	0	0	0	0	0	1	1	0	1	0	0	0	0	0	0	0	0	0	0	0	0	0	0	0	0	0	0	0	0	0	0	0	0	0	0	0	0	0	0	0	0	0	0	0	0	0	0	0	0	0	0	0	0
197.13332	ST50	103	1	1	1	1	1	1	1	1	1	1	1	1	1	1	1	1	1	1	1	1	1	1	1	1	1	1	1	1	1	1	1	1	1	1	1	1	1	1	1	1	1	1	1	1	1	1	1	1	1	1	1	1	1	1	1	1	1	1	1	1	1	1	1	1	1	1	1	1	1	1	1	1	1	1	1	1	1	1	1	1	1	1	1	1	1	1	1	1	1	1	1	1	1	1	0	0	0	0	0	0	0	0	0	0	0	0	0	0	0	0	0	0	0	0	0	0	0	0	0	0	0	0	0	0	0	0	1	1	1	1	1	1	1	0	0	0	0	0	0	0
197.4976	ST257	91	0	0	0	0	1	1	1	1	1	1	1	1	1	1	1	1	1	1	1	1	1	1	1	1	1	1	1	1	1	1	1	1	1	1	1	1	1	1	1	1	1	1	1	1	1	1	1	1	1	1	1	1	1	1	1	1	1	1	1	1	1	1	1	1	1	1	1	1	1	1	1	1	1	1	1	1	1	1	1	1	1	1	1	1	1	0	1	1	1	1	0	0	0	0	0	1	1	1	0	0	0	0	0	0	0	0	0	0	0	0	0	0	0	0	0	0	0	0	0	0	0	0	0	0	0	0	0	0	0	0	0	0	0	0	0	0	0	0	0	0
197.4991	ST257	91	0	0	0	0	1	1	1	1	1	1	1	1	1	1	1	1	1	1	1	1	1	1	1	1	1	1	1	1	1	1	1	1	1	1	1	1	1	1	1	1	1	1	1	1	1	1	1	1	1	1	1	1	1	1	1	1	1	1	1	1	1	1	1	1	1	1	1	1	1	1	1	1	1	1	1	1	1	1	1	1	1	1	1	1	1	0	1	1	1	1	0	0	0	0	0	1	1	1	0	0	0	0	0	0	0	0	0	0	0	0	0	0	0	0	0	0	0	0	0	0	0	0	0	0	0	0	0	0	0	0	0	0	0	0	0	0	0	0	0	0
197.5013	ST257	91	0	0	0	0	1	1	1	1	1	1	1	1	1	1	1	1	1	1	1	1	1	1	1	1	1	1	1	1	1	1	1	1	1	1	1	1	1	1	1	1	1	1	1	1	1	1	1	1	1	1	1	1	1	1	1	1	1	1	1	1	1	1	1	1	1	1	1	1	1	1	1	1	1	1	1	1	1	1	1	1	1	1	1	1	1	0	1	1	1	1	0	0	0	0	0	1	1	1	0	0	0	0	0	0	0	0	0	0	0	0	0	0	0	0	0	0	0	0	0	0	0	0	0	0	0	0	0	0	0	0	0	0	0	0	0	0	0	0	0	0
197.5025	ST257	91	0	0	0	0	1	1	1	1	1	1	1	1	1	1	1	1	1	1	1	1	1	1	1	1	1	1	1	1	1	1	1	1	1	1	1	1	1	1	1	1	1	1	1	1	1	1	1	1	1	1	1	1	1	1	1	1	1	1	1	1	1	1	1	1	1	1	1	1	1	1	1	1	1	1	1	1	1	1	1	1	1	1	1	1	1	0	1	1	1	1	0	0	0	0	0	1	1	1	0	0	0	0	0	0	0	0	0	0	0	0	0	0	0	0	0	0	0	0	0	0	0	0	0	0	0	0	0	0	0	0	0	0	0	0	0	0	0	0	0	0
197.5036	ST257	91	0	0	0	0	1	1	1	1	1	1	1	1	1	1	1	1	1	1	1	1	1	1	1	1	1	1	1	1	1	1	1	1	1	1	1	1	1	1	1	1	1	1	1	1	1	1	1	1	1	1	1	1	1	1	1	1	1	1	1	1	1	1	1	1	1	1	1	1	1	1	1	1	1	1	1	1	1	1	1	1	1	1	1	1	1	0	1	1	1	1	0	0	0	0	0	1	1	1	0	0	0	0	0	0	0	0	0	0	0	0	0	0	0	0	0	0	0	0	0	0	0	0	0	0	0	0	0	0	0	0	0	0	0	0	0	0	0	0	0	0
197.5065	ST257	91	0	0	0	0	1	1	1	1	1	1	1	1	1	1	1	1	1	1	1	1	1	1	1	1	1	1	1	1	1	1	1	1	1	1	1	1	1	1	1	1	1	1	1	1	1	1	1	1	1	1	1	1	1	1	1	1	1	1	1	1	1	1	1	1	1	1	1	1	1	1	1	1	1	1	1	1	1	1	1	1	1	1	1	1	1	0	1	1	1	1	0	0	0	0	0	1	1	1	0	0	0	0	0	0	0	0	0	0	0	0	0	0	0	0	0	0	0	0	0	0	0	0	0	0	0	0	0	0	0	0	0	0	0	0	0	0	0	0	0	0
197.5106	ST257	91	0	0	0	0	1	1	1	1	1	1	1	1	1	1	1	1	1	1	1	1	1	1	1	1	1	1	1	1	1	1	1	1	1	1	1	1	1	1	1	1	1	1	1	1	1	1	1	1	1	1	1	1	1	1	1	1	1	1	1	1	1	1	1	1	1	1	1	1	1	1	1	1	1	1	1	1	1	1	1	1	1	1	1	1	1	0	1	1	1	1	0	1	0	0	0	1	1	1	0	0	0	0	0	0	0	0	0	0	0	0	0	0	0	0	0	0	0	0	0	0	0	0	0	0	0	0	0	0	0	0	0	0	0	0	0	0	0	0	0	0
197.5257	ST257	91	0	0	0	0	1	1	1	1	1	1	1	1	1	1	1	1	1	1	1	1	1	1	1	1	1	1	1	1	1	1	1	1	1	1	1	1	1	1	1	1	1	1	1	1	1	1	1	1	1	1	1	1	1	1	1	1	1	1	1	1	1	1	1	1	1	1	1	1	1	1	1	1	1	1	1	1	1	1	1	1	1	1	1	1	1	0	1	1	1	1	0	1	0	0	0	1	1	1	0	0	0	0	0	0	0	0	0	0	0	0	0	0	0	0	0	0	0	0	0	0	0	0	0	0	0	0	0	0	0	0	0	0	0	0	0	0	0	0	0	0
197.4839	ST257	92	0	0	0	0	1	1	1	1	1	1	1	1	1	1	1	1	1	1	1	1	1	1	1	1	1	1	1	1	1	1	1	1	1	1	1	1	1	1	1	1	1	1	1	1	1	1	1	1	1	1	1	1	1	1	1	1	1	1	1	1	1	1	1	1	1	1	1	1	1	1	1	1	1	1	1	1	1	1	1	1	1	1	1	1	1	0	1	1	1	1	0	1	0	0	0	1	1	1	0	0	0	0	0	0	0	0	0	0	0	0	0	0	0	0	0	0	0	0	0	0	0	0	0	0	0	0	0	0	0	0	0	0	0	0	0	0	0	0	0	0
197.4860	ST257	92	0	0	0	0	1	1	1	1	1	1	1	1	1	1	1	1	1	1	1	1	1	1	1	1	1	1	1	1	1	1	1	1	1	1	1	1	1	1	1	1	1	1	1	1	1	1	1	1	1	1	1	1	1	1	1	1	1	1	1	1	1	1	1	1	1	1	1	1	1	1	1	1	1	1	1	1	1	1	1	1	1	1	1	1	1	0	1	1	1	1	0	1	1	0	0	1	1	1	0	0	0	0	0	0	0	0	0	0	0	0	0	0	0	0	0	0	0	0	0	0	0	0	0	0	0	0	0	0	0	0	0	0	0	0	0	0	0	0	0	0
197.5258	ST257	92	0	0	0	0	1	1	1	1	1	1	1	1	1	1	1	1	1	1	1	1	1	1	1	1	1	1	1	1	1	1	1	1	1	1	1	1	1	1	1	1	1	1	1	1	1	1	1	1	1	1	1	1	1	1	1	1	1	1	1	1	1	1	1	1	1	1	1	1	1	1	1	1	1	1	1	1	1	1	1	1	1	1	1	1	1	0	1	1	1	1	0	1	1	0	0	1	1	1	0	0	0	0	0	0	0	0	0	0	0	0	0	0	0	0	0	0	0	0	0	0	0	0	0	0	0	0	0	0	0	0	0	0	0	0	0	0	0	0	0	0
197.6740	ST257	92	0	0	0	0	1	1	1	1	1	1	1	1	1	1	1	1	1	1	1	1	1	1	1	1	1	1	1	1	1	1	1	1	1	1	1	1	1	1	1	1	1	1	1	1	1	1	1	1	1	1	1	1	1	1	1	1	1	1	1	1	1	1	1	1	1	1	1	1	1	1	1	1	1	1	1	1	1	1	1	1	1	1	1	1	1	0	1	1	1	1	0	1	1	0	0	1	1	1	0	0	0	0	0	0	0	0	0	0	0	0	0	0	0	0	0	0	0	0	0	0	0	0	0	0	0	0	0	0	0	0	0	0	0	0	0	0	0	0	0	0
197.4973	ST257	93	0	0	0	0	1	1	1	1	1	1	1	1	1	1	1	1	1	1	1	1	1	1	1	1	1	1	1	1	1	1	1	1	1	1	1	1	1	1	1	1	1	1	1	1	1	1	1	1	1	1	1	1	1	1	1	1	1	1	1	1	1	1	1	1	1	1	1	1	1	1	1	1	1	1	1	1	1	1	1	1	1	1	1	1	1	0	1	1	1	1	0	0	1	0	0	1	1	1	0	0	0	0	0	0	0	0	0	0	0	0	0	0	0	0	0	0	0	0	0	0	0	0	0	0	0	0	0	0	0	0	0	0	0	0	0	0	0	0	0	0
197.5006	ST257	93	0	0	0	0	1	1	1	1	1	1	1	1	1	1	1	1	1	1	1	1	1	1	1	1	1	1	1	1	1	1	1	1	1	1	1	1	1	1	1	1	1	1	1	1	1	1	1	1	1	1	1	1	1	1	1	1	1	1	1	1	1	1	1	1	1	1	1	1	1	1	1	1	1	1	1	1	1	1	1	1	1	1	1	1	1	0	1	1	1	1	0	0	1	0	0	1	1	1	0	0	0	0	0	0	0	0	0	0	0	0	0	0	0	0	0	0	0	0	0	0	0	0	0	0	0	0	0	0	0	0	0	0	0	0	0	0	0	0	0	0
197.7960	ST257	93	0	0	0	0	1	1	1	1	1	1	1	1	1	1	1	1	1	1	1	1	1	1	1	1	1	1	1	1	1	1	1	1	1	1	1	1	1	1	1	1	1	1	1	1	1	1	1	1	1	1	1	1	1	1	1	1	1	1	1	1	1	1	1	1	1	1	1	1	1	1	1	1	1	1	1	1	1	1	1	1	1	1	1	1	1	0	1	1	1	1	0	0	1	0	0	1	1	1	0	0	0	0	0	0	0	0	0	0	0	0	0	0	0	0	0	0	0	0	0	0	0	0	0	0	0	0	0	0	0	0	0	0	0	0	0	0	0	0	0	0
197.4930	ST257	92	0	0	0	0	1	1	1	1	1	1	1	1	1	1	1	1	1	1	1	1	1	1	1	1	1	1	1	1	1	1	1	1	1	1	1	1	1	1	1	1	1	1	1	1	1	1	1	1	1	1	1	1	1	1	1	1	1	1	1	1	1	1	1	1	1	1	1	1	1	1	1	1	1	1	1	1	1	1	1	1	1	1	1	1	1	0	1	1	1	1	0	0	0	0	0	1	0	1	0	0	0	0	0	0	0	0	0	0	0	0	0	0	0	0	0	0	0	0	0	0	0	0	0	0	0	0	0	0	0	0	0	0	0	0	0	0	0	0	0	0
197.5015	ST257	92	0	0	0	0	1	1	1	1	1	1	1	1	1	1	1	1	1	1	1	1	1	1	1	1	1	1	1	1	1	1	1	1	1	1	1	1	1	1	1	1	1	1	1	1	1	1	1	1	1	1	1	1	1	1	1	1	1	1	1	1	1	1	1	1	1	1	1	1	1	1	1	1	1	1	1	1	1	1	1	1	1	1	1	1	1	0	1	1	1	1	0	0	0	0	0	0	0	1	0	0	0	0	0	0	0	0	0	0	0	0	0	0	0	0	0	0	0	0	0	0	0	0	0	0	0	0	0	0	0	0	0	0	0	0	0	0	0	0	0	0
197.5050	ST257	89	0	0	0	0	1	1	1	1	1	1	1	1	1	1	1	1	1	1	1	1	1	1	1	1	1	1	1	1	1	1	1	1	1	1	1	1	1	1	1	1	1	1	1	1	1	1	1	1	1	1	1	1	1	1	1	1	1	1	1	1	1	1	1	1	1	1	1	1	1	1	1	1	1	1	1	1	1	1	1	1	1	1	1	1	1	0	1	1	1	1	0	0	0	0	0	0	0	1	0	0	0	0	0	0	0	0	0	0	0	0	0	0	0	0	0	0	0	0	0	0	0	0	0	0	0	0	0	0	0	0	0	0	0	0	0	0	0	0	0	0
KF045	ST257	89	0	0	0	0	1	1	1	1	1	1	1	1	1	1	1	1	1	1	1	1	1	1	1	1	1	1	1	1	1	1	1	1	1	1	1	1	1	1	1	1	1	1	1	1	1	1	1	1	1	1	1	1	1	1	1	1	1	1	1	1	1	1	1	1	1	1	1	1	1	1	1	1	1	1	1	1	1	1	1	1	1	1	1	1	1	0	1	1	1	1	0	0	0	0	0	1	1	1	0	0	0	0	0	0	0	0	0	0	0	0	0	0	0	0	0	0	0	0	0	0	0	0	0	0	0	0	0	0	0	0	0	0	0	0	0	0	0	0	0	0
1248402.4	ST257	80	0	0	0	0	1	1	1	1	1	1	1	1	1	1	1	1	1	1	1	1	1	1	1	1	1	1	1	1	1	1	1	1	1	1	1	1	1	1	1	1	1	1	1	1	1	1	1	1	1	1	1	1	1	1	1	1	1	1	1	1	1	1	1	1	1	1	1	1	1	1	1	1	1	1	0	0	0	0	1	1	1	1	1	0	0	0	0	1	1	1	0	0	0	0	0	0	0	0	0	0	0	0	0	0	0	0	0	0	0	0	0	0	0	0	0	0	0	0	0	0	0	0	0	0	0	0	0	0	0	0	0	0	0	0	0	0	0	0	0	0
197.11657	ST50	105	1	1	1	1	1	1	1	1	1	1	1	1	1	1	1	1	1	1	1	1	1	1	1	1	1	1	1	1	1	1	1	1	1	1	1	1	1	1	1	1	1	1	1	1	1	1	1	1	1	1	1	1	1	1	1	1	1	1	1	1	1	1	1	1	1	1	1	1	1	1	1	1	1	1	1	1	1	1	1	1	1	1	1	1	1	0	1	1	1	1	1	1	0	0	0	0	1	1	0	0	0	0	0	0	0	0	0	0	0	0	0	0	0	0	0	0	1	1	1	1	1	1	1	1	1	1	0	0	0	0	0	0	0	0	0	0	0	0	0	0	0
197.5328	ST50	107	1	1	1	1	1	1	1	1	1	1	1	1	1	1	1	1	1	1	1	1	1	1	1	1	1	1	1	1	1	1	1	1	1	1	1	1	1	1	1	1	1	1	1	1	1	1	1	1	1	1	1	1	1	1	1	1	1	1	1	1	1	1	1	1	1	1	1	1	1	1	1	1	1	1	1	1	1	1	1	1	1	1	1	1	1	1	1	1	1	1	1	1	0	0	0	0	1	1	0	0	0	0	0	0	0	0	0	0	0	0	0	0	0	0	0	0	1	1	1	1	1	1	1	1	1	1	1	0	0	0	0	0	0	0	0	0	0	0	0	0	0
197.5327	ST50	115	1	1	1	1	1	1	1	1	1	1	1	1	1	1	1	1	1	1	1	1	1	1	1	1	1	1	1	1	1	1	1	1	1	1	1	1	1	1	1	1	1	1	1	1	1	1	1	1	1	1	1	1	1	1	1	1	1	1	1	1	1	1	1	1	1	1	1	1	1	1	1	1	1	1	1	1	1	1	1	1	1	0	1	1	1	1	1	1	1	1	1	1	1	1	0	0	1	1	0	0	0	0	0	0	0	0	0	0	0	0	0	0	0	0	0	0	1	1	1	1	1	1	1	1	1	1	1	1	1	1	1	1	1	1	0	0	0	0	0	0	0
197.4804	ST50	111	1	1	1	1	1	1	1	1	1	1	1	1	1	1	1	1	1	1	1	1	1	1	1	1	1	1	1	1	1	1	1	1	1	1	1	1	1	1	1	1	1	1	1	1	1	1	1	1	1	1	1	1	1	1	1	1	1	1	1	1	1	1	1	1	1	1	1	1	1	1	1	1	1	1	1	1	1	1	1	1	1	1	1	1	1	1	0	1	1	1	1	1	1	1	0	0	0	0	0	1	1	1	1	1	1	1	1	1	1	1	1	1	1	1	1	0	0	0	0	0	0	0	0	0	0	0	0	0	0	0	0	0	0	0	0	0	0	0	0	0
197.4805	ST50	111	1	1	1	1	1	1	1	1	1	1	1	1	1	1	1	1	1	1	1	1	1	1	1	1	1	1	1	1	1	1	1	1	1	1	1	1	1	1	1	1	1	1	1	1	1	1	1	1	1	1	1	1	1	1	1	1	1	1	1	1	1	1	1	1	1	1	1	1	1	1	1	1	1	1	1	1	1	1	1	1	1	1	1	1	1	1	0	1	1	1	1	1	1	1	0	0	0	0	0	1	1	1	1	1	1	1	1	1	1	1	1	1	1	1	1	0	0	0	0	0	0	0	0	0	0	0	0	0	0	0	0	0	0	0	0	0	0	0	0	0
197.4806	ST50	111	1	1	1	1	1	1	1	1	1	1	1	1	1	1	1	1	1	1	1	1	1	1	1	1	1	1	1	1	1	1	1	1	1	1	1	1	1	1	1	1	1	1	1	1	1	1	1	1	1	1	1	1	1	1	1	1	1	1	1	1	1	1	1	1	1	1	1	1	1	1	1	1	1	1	1	1	1	1	1	1	1	1	1	1	1	1	0	1	1	1	1	1	1	1	0	0	0	0	0	1	1	1	1	1	1	1	1	1	1	1	1	1	1	1	1	0	0	0	0	0	0	0	0	0	0	0	0	0	0	0	0	0	0	0	0	0	0	0	0	0
197.4810	ST50	112	1	1	1	1	1	1	1	1	1	1	1	1	1	1	1	1	1	1	1	1	1	1	1	1	1	1	1	1	1	1	1	1	1	1	1	1	1	1	1	1	1	1	1	1	1	1	1	1	1	1	1	1	1	1	1	1	1	1	1	1	1	1	1	1	1	1	1	1	1	1	1	1	1	1	1	1	1	1	1	1	1	1	1	1	1	1	0	1	1	1	1	1	1	1	0	0	0	0	0	1	1	1	1	1	1	1	1	1	1	1	1	1	1	1	1	1	0	0	0	0	0	0	0	0	0	0	0	0	0	0	0	0	0	0	0	0	0	0	0	0
197.4807	ST50	110	1	1	1	1	1	1	1	1	1	1	1	1	1	1	1	1	1	1	1	1	1	1	1	1	1	1	1	1	1	1	1	1	1	1	1	1	1	1	1	1	1	1	1	1	1	1	1	1	1	1	1	1	1	1	1	1	1	1	1	1	1	1	1	1	1	1	1	1	1	1	1	1	1	1	1	1	1	1	1	1	1	1	1	1	1	1	0	1	1	1	1	1	0	1	0	0	0	0	0	1	1	1	1	1	1	1	1	1	1	1	1	1	1	1	1	0	0	0	0	0	0	0	0	0	0	0	0	0	0	0	0	0	0	0	0	0	0	0	0	0
197.4809	ST50	110	1	1	1	1	1	1	1	1	1	1	1	1	1	1	1	1	1	1	1	1	1	1	1	1	1	1	1	1	1	1	1	1	1	1	1	1	1	1	1	1	1	1	1	1	1	1	1	1	1	1	1	1	1	1	1	1	1	1	1	1	1	1	1	1	1	1	1	1	1	1	1	1	1	1	1	1	1	1	1	1	1	1	1	1	1	1	0	1	1	1	1	1	1	1	0	0	0	0	0	1	1	1	1	1	1	1	1	1	1	1	1	1	0	1	1	0	0	0	0	0	0	0	0	0	0	0	0	0	0	0	0	0	0	0	0	0	0	0	0	0

Table S5

The occurence of 25 genes recognized by Buchanan et al. [17] as markers of human pathogenic *C. jejuni* strains in genomes of *C. jejuni* ST50, ST51 and ST257.

Genome ID	ST	Number of genes	Cj0055c	Cj0056c	Cj0177	Cj0178	Cj0179	Cj0180	Cj0181	Cj0246c	Cj0259	Cj0260c	Cj0264c	Cj0265c	Cj0295	Cj0297c	Cj0296c	Cj0298c	Cj0299	Cj0731	Cj0753c	Cj0755	Cj1122c	Cj1255	Cj1365c	Cj1585c	Cj1679
197.4908	ST50	23	1	1	1	1	1	1	1	1	1	1	1	1	0	1	1	1	1	1	1	1	1	0	1	1	1
197.4933	ST50	23	1	1	1	1	1	1	1	1	1	1	1	1	0	1	1	1	1	1	1	1	1	0	1	1	1
197.5072	ST50	23	1	1	1	1	1	1	1	1	1	1	1	1	0	1	1	1	1	1	1	1	1	0	1	1	1
197.5110	ST50	23	1	1	1	1	1	1	1	1	1	1	1	1	0	1	1	1	1	1	1	1	1	0	1	1	1
1201032.3	ST50	24	1	1	1	1	1	1	1	1	1	1	1	1	1	1	1	1	1	1	1	1	1	0	1	1	1
197.11580	ST50	24	1	1	1	1	1	1	1	1	1	1	1	1	1	1	1	1	1	1	1	1	1	0	1	1	1
197.11657	ST50	24	1	1	1	1	1	1	1	1	1	1	1	1	1	1	1	1	1	1	1	1	1	0	1	1	1
197.13041	ST50	24	1	1	1	1	1	1	1	1	1	1	1	1	1	1	1	1	1	1	1	1	1	0	1	1	1
197.13052	ST50	24	1	1	1	1	1	1	1	1	1	1	1	1	1	1	1	1	1	1	1	1	1	0	1	1	1
197.13332	ST50	24	1	1	1	1	1	1	1	1	1	1	1	1	1	1	1	1	1	1	1	1	1	0	1	1	1
197.4804	ST50	24	1	1	1	1	1	1	1	1	1	1	1	1	1	1	1	1	1	1	1	1	1	0	1	1	1
197.4805	ST50	24	1	1	1	1	1	1	1	1	1	1	1	1	1	1	1	1	1	1	1	1	1	0	1	1	1
197.4806	ST50	24	1	1	1	1	1	1	1	1	1	1	1	1	1	1	1	1	1	1	1	1	1	0	1	1	1
197.4807	ST50	24	1	1	1	1	1	1	1	1	1	1	1	1	1	1	1	1	1	1	1	1	1	0	1	1	1
197.4808	ST50	24	1	1	1	1	1	1	1	1	1	1	1	1	1	1	1	1	1	1	1	1	1	0	1	1	1
197.4809	ST50	24	1	1	1	1	1	1	1	1	1	1	1	1	1	1	1	1	1	1	1	1	1	0	1	1	1
197.4810	ST50	24	1	1	1	1	1	1	1	1	1	1	1	1	1	1	1	1	1	1	1	1	1	0	1	1	1
197.4824	ST50	24	1	1	1	1	1	1	1	1	1	1	1	1	1	1	1	1	1	1	1	1	1	0	1	1	1
197.4925	ST50	24	1	1	1	1	1	1	1	1	1	1	1	1	1	1	1	1	1	1	1	1	1	0	1	1	1
197.4950	ST50	24	1	1	1	1	1	1	1	1	1	1	1	1	1	1	1	1	1	1	1	1	1	0	1	1	1
197.4952	ST50	24	1	1	1	1	1	1	1	1	1	1	1	1	1	1	1	1	1	1	1	1	1	0	1	1	1
197.4983	ST50	24	1	1	1	1	1	1	1	1	1	1	1	1	1	1	1	1	1	1	1	1	1	0	1	1	1
197.5003	ST50	24	1	1	1	1	1	1	1	1	1	1	1	1	1	1	1	1	1	1	1	1	1	0	1	1	1
197.5020	ST50	24	1	1	1	1	1	1	1	1	1	1	1	1	1	1	1	1	1	1	1	1	1	0	1	1	1
197.5026	ST50	24	1	1	1	1	1	1	1	1	1	1	1	1	1	1	1	1	1	1	1	1	1	0	1	1	1
197.5035	ST50	24	1	1	1	1	1	1	1	1	1	1	1	1	1	1	1	1	1	1	1	1	1	0	1	1	1
197.5074	ST50	24	1	1	1	1	1	1	1	1	1	1	1	1	1	1	1	1	1	1	1	1	1	0	1	1	1
197.5099	ST50	24	1	1	1	1	1	1	1	1	1	1	1	1	1	1	1	1	1	1	1	1	1	0	1	1	1
197.5113	ST50	24	1	1	1	1	1	1	1	1	1	1	1	1	1	1	1	1	1	1	1	1	1	0	1	1	1
197.5165	ST50	24	1	1	1	1	1	1	1	1	1	1	1	1	1	1	1	1	1	1	1	1	1	0	1	1	1
197.5328	ST50	24	1	1	1	1	1	1	1	1	1	1	1	1	1	1	1	1	1	1	1	1	1	0	1	1	1
197.6704	ST50	24	1	1	1	1	1	1	1	1	1	1	1	1	1	1	1	1	1	1	1	1	1	0	1	1	1
197.6746	ST50	24	1	1	1	1	1	1	1	1	1	1	1	1	1	1	1	1	1	1	1	1	1	0	1	1	1
197.6758	ST50	24	1	1	1	1	1	1	1	1	1	1	1	1	1	1	1	1	1	1	1	1	1	0	1	1	1
32022.274	ST50	24	1	1	1	1	1	1	1	1	1	1	1	1	1	1	1	1	1	1	1	1	1	0	1	1	1
32022.276	ST50	24	1	1	1	1	1	1	1	1	1	1	1	1	1	1	1	1	1	1	1	1	1	0	1	1	1
32022.279	ST50	24	1	1	1	1	1	1	1	1	1	1	1	1	1	1	1	1	1	1	1	1	1	0	1	1	1
32022.280	ST50	24	1	1	1	1	1	1	1	1	1	1	1	1	1	1	1	1	1	1	1	1	1	0	1	1	1
32022.281	ST50	24	1	1	1	1	1	1	1	1	1	1	1	1	1	1	1	1	1	1	1	1	1	0	1	1	1
32022.282	ST50	24	1	1	1	1	1	1	1	1	1	1	1	1	1	1	1	1	1	1	1	1	1	0	1	1	1
32022.283	ST50	24	1	1	1	1	1	1	1	1	1	1	1	1	1	1	1	1	1	1	1	1	1	0	1	1	1
32022.284	ST50	24	1	1	1	1	1	1	1	1	1	1	1	1	1	1	1	1	1	1	1	1	1	0	1	1	1
32022.285	ST50	24	1	1	1	1	1	1	1	1	1	1	1	1	1	1	1	1	1	1	1	1	1	0	1	1	1
32022.286	ST50	24	1	1	1	1	1	1	1	1	1	1	1	1	1	1	1	1	1	1	1	1	1	0	1	1	1
32022.287	ST50	24	1	1	1	1	1	1	1	1	1	1	1	1	1	1	1	1	1	1	1	1	1	0	1	1	1
32022.288	ST50	24	1	1	1	1	1	1	1	1	1	1	1	1	1	1	1	1	1	1	1	1	1	0	1	1	1
32022.290	ST50	24	1	1	1	1	1	1	1	1	1	1	1	1	1	1	1	1	1	1	1	1	1	0	1	1	1
32022.634	ST50	24	1	1	1	1	1	1	1	1	1	1	1	1	1	1	1	1	1	1	1	1	1	0	1	1	1
889228.3	ST50	24	1	1	1	1	1	1	1	1	1	1	1	1	1	1	1	1	1	1	1	1	1	0	1	1	1
889250.3	ST50	24	1	1	1	1	1	1	1	1	1	1	1	1	1	1	1	1	1	1	1	1	1	0	1	1	1
KF017	ST50	24	1	1	1	1	1	1	1	1	1	1	1	1	1	1	1	1	1	1	1	1	1	0	1	1	1
KF042	ST50	24	1	1	1	1	1	1	1	1	1	1	1	1	1	1	1	1	1	1	1	1	1	0	1	1	1
197.5184	ST50	22	1	1	1	1	1	1	1	1	1	1	1	1	0	1	1	1	1	0	1	1	1	0	1	1	1
197.5228	ST50	22	1	1	1	1	1	1	1	1	1	1	1	1	0	1	1	1	1	0	1	1	1	0	1	1	1
889255.3	ST50	22	1	1	1	1	1	1	1	1	1	1	1	1	0	1	1	1	1	0	1	1	1	0	1	1	1
197.6730	ST50	24	1	1	1	1	1	1	1	1	1	1	1	1	1	1	1	1	1	1	1	1	0	1	1	1	1
197.4814	ST50	21	1	1	1	1	1	1	1	1	1	0	0	0	1	1	1	1	1	1	1	1	1	0	1	1	1
197.4816	ST50	21	1	1	1	1	1	1	1	1	1	0	0	0	1	1	1	1	1	1	1	1	1	0	1	1	1
197.4838	ST50	21	1	1	1	1	1	1	1	1	1	0	0	0	1	1	1	1	1	1	1	1	1	0	1	1	1
197.4871	ST50	21	1	1	1	1	1	1	1	1	1	0	0	0	1	1	1	1	1	1	1	1	1	0	1	1	1
197.4901	ST50	21	1	1	1	1	1	1	1	1	1	0	0	0	1	1	1	1	1	1	1	1	1	0	1	1	1
197.4910	ST50	21	1	1	1	1	1	1	1	1	1	0	0	0	1	1	1	1	1	1	1	1	1	0	1	1	1
197.5001	ST50	21	1	1	1	1	1	1	1	1	1	0	0	0	1	1	1	1	1	1	1	1	1	0	1	1	1
197.5004	ST50	21	1	1	1	1	1	1	1	1	1	0	0	0	1	1	1	1	1	1	1	1	1	0	1	1	1
197.5038	ST50	21	1	1	1	1	1	1	1	1	1	0	0	0	1	1	1	1	1	1	1	1	1	0	1	1	1
197.5076	ST50	21	1	1	1	1	1	1	1	1	1	0	0	0	1	1	1	1	1	1	1	1	1	0	1	1	1
197.5092	ST50	21	1	1	1	1	1	1	1	1	1	0	0	0	1	1	1	1	1	1	1	1	1	0	1	1	1
197.5133	ST50	21	1	1	1	1	1	1	1	1	1	0	0	0	1	1	1	1	1	1	1	1	1	0	1	1	1
1440029.3	ST50	22	1	1	1	1	1	1	1	1	1	1	0	0	1	1	1	1	1	1	1	1	1	0	1	1	1
1440034.3	ST50	22	1	1	1	1	1	1	1	1	1	1	0	0	1	1	1	1	1	1	1	1	1	0	1	1	1
197.5327	ST50	20	0	0	1	1	1	1	1	1	1	0	1	1	0	1	1	1	1	1	1	1	1	0	1	1	1
197.4827	ST51	17	0	0	1	1	1	1	1	0	1	1	0	0	1	1	1	1	1	1	1	1	0	0	1	1	0
197.4864	ST51	17	0	0	1	1	1	1	1	0	1	1	0	0	1	1	1	1	1	1	1	1	0	0	1	1	0
197.4870	ST51	17	0	0	1	1	1	1	1	0	1	1	0	0	1	1	1	1	1	1	1	1	0	0	1	1	0
197.4975	ST51	17	0	0	1	1	1	1	1	0	1	1	0	0	1	1	1	1	1	1	1	1	0	0	1	1	0
197.5069	ST51	17	0	0	1	1	1	1	1	0	1	1	0	0	1	1	1	1	1	1	1	1	0	0	1	1	0
197.5088	ST51	17	0	0	1	1	1	1	1	0	1	1	0	0	1	1	1	1	1	1	1	1	0	0	1	1	0
197.5137	ST51	17	0	0	1	1	1	1	1	0	1	1	0	0	1	1	1	1	1	1	1	1	0	0	1	1	0
197.7745	ST51	17	0	0	1	1	1	1	1	0	1	1	0	0	1	1	1	1	1	1	1	1	0	0	1	1	0
1248402.4	ST257	17	0	0	1	1	1	1	1	0	1	1	0	0	1	1	1	1	1	1	1	1	1	0	1	0	0
197.4839	ST257	17	0	0	1	1	1	1	1	0	1	1	0	0	1	1	1	1	1	1	1	1	1	0	1	0	0
197.4860	ST257	17	0	0	1	1	1	1	1	0	1	1	0	0	1	1	1	1	1	1	1	1	1	0	1	0	0
197.4930	ST257	17	0	0	1	1	1	1	1	0	1	1	0	0	1	1	1	1	1	1	1	1	1	0	1	0	0
197.4973	ST257	17	0	0	1	1	1	1	1	0	1	1	0	0	1	1	1	1	1	1	1	1	1	0	1	0	0
197.4976	ST257	17	0	0	1	1	1	1	1	0	1	1	0	0	1	1	1	1	1	1	1	1	1	0	1	0	0
197.4991	ST257	17	0	0	1	1	1	1	1	0	1	1	0	0	1	1	1	1	1	1	1	1	1	0	1	0	0
197.5006	ST257	17	0	0	1	1	1	1	1	0	1	1	0	0	1	1	1	1	1	1	1	1	1	0	1	0	0
197.5013	ST257	17	0	0	1	1	1	1	1	0	1	1	0	0	1	1	1	1	1	1	1	1	1	0	1	0	0
197.5015	ST257	17	0	0	1	1	1	1	1	0	1	1	0	0	1	1	1	1	1	1	1	1	1	0	1	0	0
197.5025	ST257	17	0	0	1	1	1	1	1	0	1	1	0	0	1	1	1	1	1	1	1	1	1	0	1	0	0
197.5036	ST257	17	0	0	1	1	1	1	1	0	1	1	0	0	1	1	1	1	1	1	1	1	1	0	1	0	0
197.5050	ST257	17	0	0	1	1	1	1	1	0	1	1	0	0	1	1	1	1	1	1	1	1	1	0	1	0	0
197.5065	ST257	17	0	0	1	1	1	1	1	0	1	1	0	0	1	1	1	1	1	1	1	1	1	0	1	0	0
197.5106	ST257	17	0	0	1	1	1	1	1	0	1	1	0	0	1	1	1	1	1	1	1	1	1	0	1	0	0
197.5257	ST257	17	0	0	1	1	1	1	1	0	1	1	0	0	1	1	1	1	1	1	1	1	1	0	1	0	0
197.5258	ST257	17	0	0	1	1	1	1	1	0	1	1	0	0	1	1	1	1	1	1	1	1	1	0	1	0	0
197.6740	ST257	17	0	0	1	1	1	1	1	0	1	1	0	0	1	1	1	1	1	1	1	1	1	0	1	0	0
197.7960	ST257	17	0	0	1	1	1	1	1	0	1	1	0	0	1	1	1	1	1	1	1	1	1	0	1	0	0
KF045	ST257	17	0	0	1	1	1	1	1	0	1	1	0	0	1	1	1	1	1	1	1	1	1	0	1	0	0
197.5167	ST51	11	0	0	1	1	1	1	1	0	1	1	0	0	0	0	0	0	0	0	1	1	0	0	1	1	0
197.5174	ST51	11	0	0	1	1	1	1	1	0	1	1	0	0	0	0	0	0	0	0	1	1	0	0	1	1	0
1316917.3	ST51	12	0	0	1	1	1	1	1	0	1	1	0	0	0	0	0	0	0	1	1	1	0	0	1	1	0
1321759.3	ST51	12	0	0	1	1	1	1	1	0	1	1	0	0	0	0	0	0	0	1	1	1	0	0	1	1	0
197.11671	ST51	12	0	0	1	1	1	1	1	0	1	1	0	0	0	0	0	0	0	1	1	1	0	0	1	1	0
197.4867	ST51	12	0	0	1	1	1	1	1	0	1	1	0	0	0	0	0	0	0	1	1	1	0	0	1	1	0
197.4956	ST51	12	0	0	1	1	1	1	1	0	1	1	0	0	0	0	0	0	0	1	1	1	0	0	1	1	0
197.4994	ST51	12	0	0	1	1	1	1	1	0	1	1	0	0	0	0	0	0	0	1	1	1	0	0	1	1	0
197.5010	ST51	12	0	0	1	1	1	1	1	0	1	1	0	0	0	0	0	0	0	1	1	1	0	0	1	1	0
197.5043	ST51	12	0	0	1	1	1	1	1	0	1	1	0	0	0	0	0	0	0	1	1	1	0	0	1	1	0
197.5185	ST51	12	0	0	1	1	1	1	1	0	1	1	0	0	0	0	0	0	0	1	1	1	0	0	1	1	0
197.7920	ST51	12	0	0	1	1	1	1	1	0	1	1	0	0	0	0	0	0	0	1	1	1	0	0	1	1	0
197.7921	ST51	12	0	0	1	1	1	1	1	0	1	1	0	0	0	0	0	0	0	1	1	1	0	0	1	1	0
32022.608	ST51	12	0	0	1	1	1	1	1	0	1	1	0	0	0	0	0	0	0	1	1	1	0	0	1	1	0
32022.609	ST51	12	0	0	1	1	1	1	1	0	1	1	0	0	0	0	0	0	0	1	1	1	0	0	1	1	0
32022.610	ST51	12	0	0	1	1	1	1	1	0	1	1	0	0	0	0	0	0	0	1	1	1	0	0	1	1	0
KF070	ST51	12	0	0	1	1	1	1	1	0	1	1	0	0	0	0	0	0	0	1	1	1	0	0	1	1	0

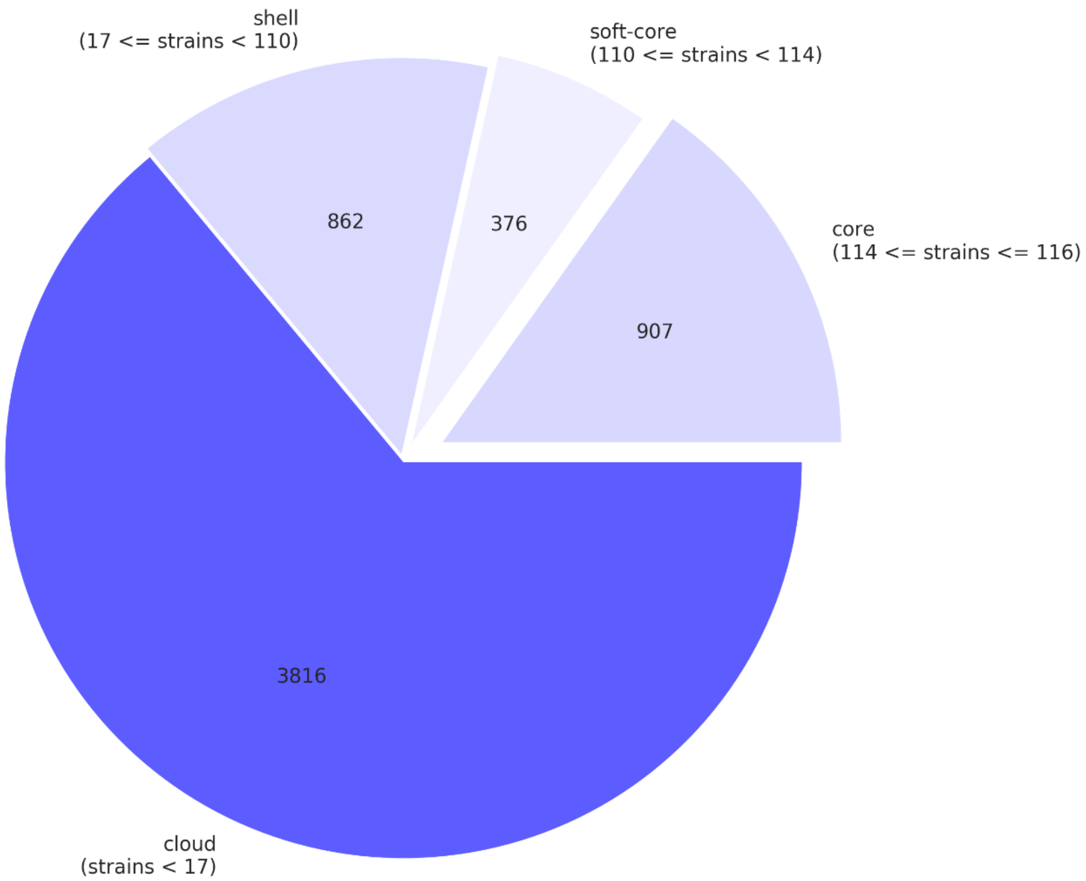

Fig. S1 The distribution *C. jejuni* pan-genome genes and the number of isolates possessing them (visualized using roary_plots.py script).

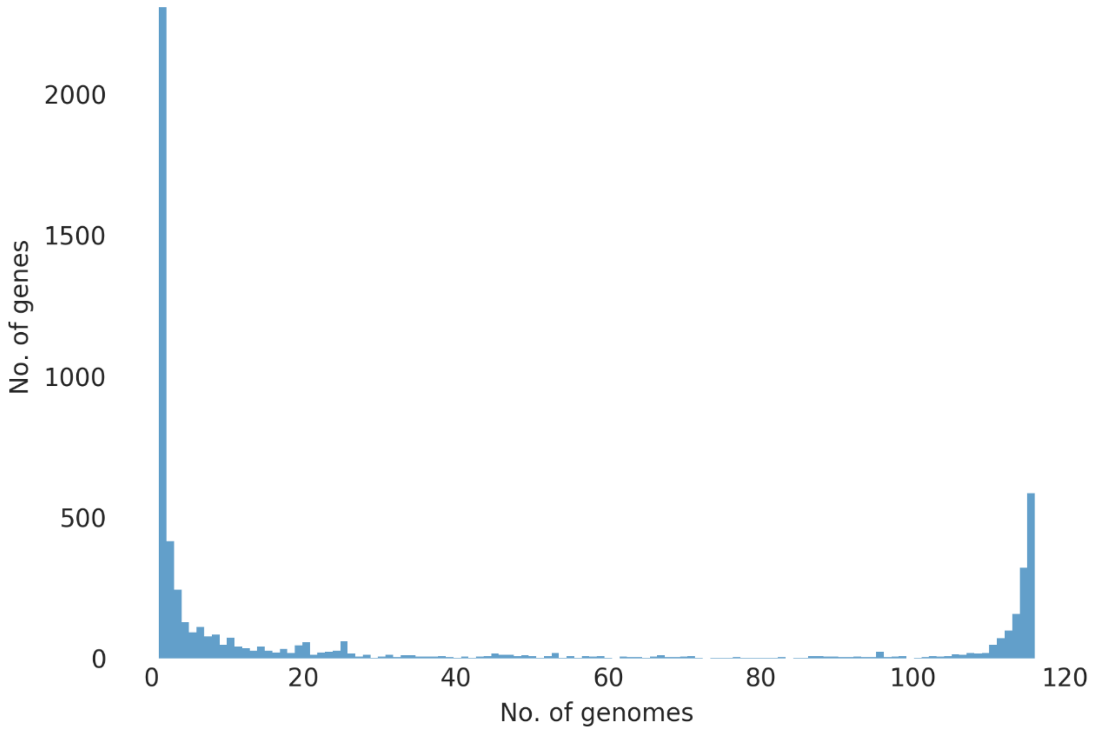

Fig. S2 The frequency of *C. jejuni* genes versus the number of genomes (visualized using roary_plots.py script).

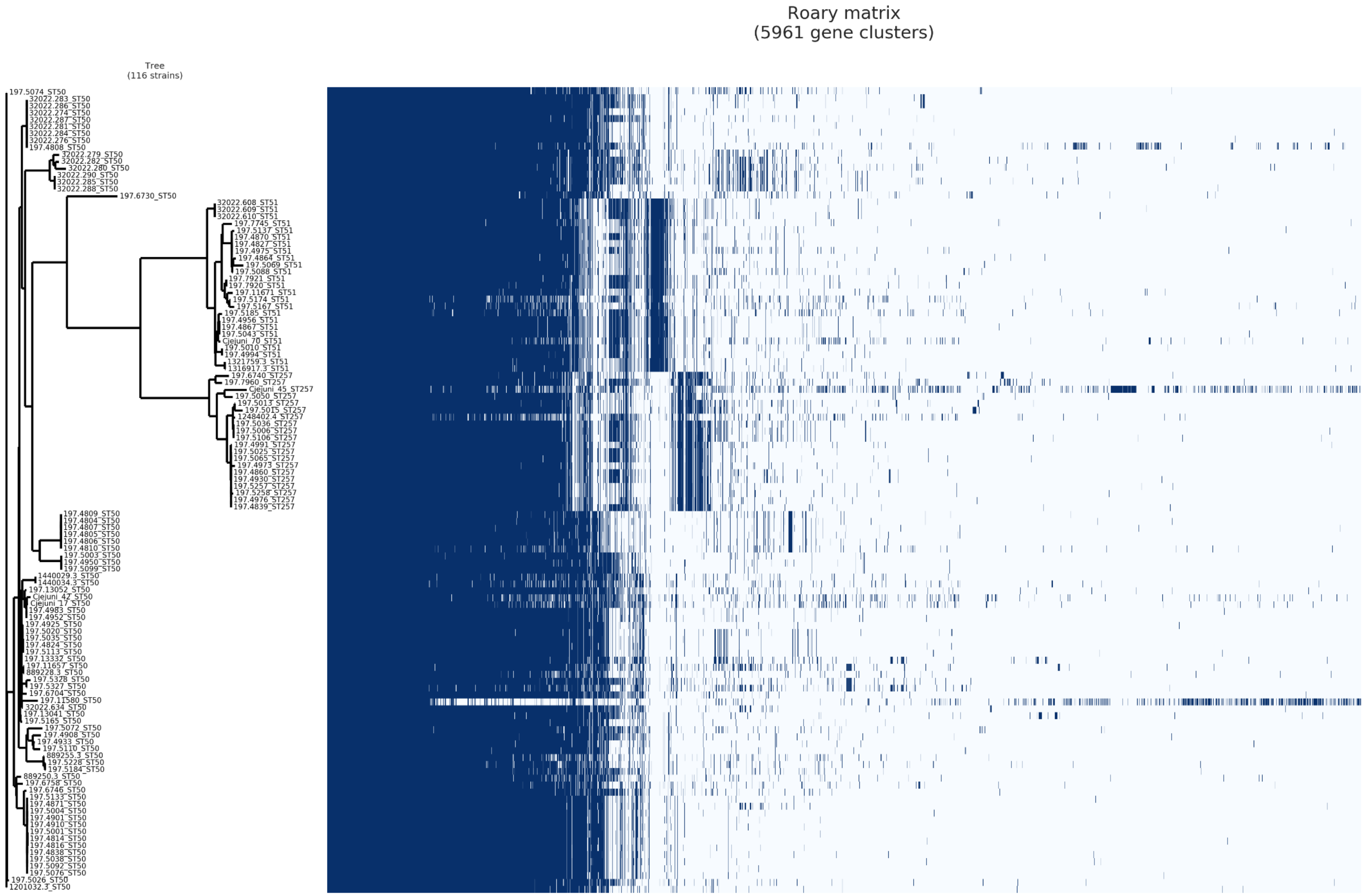

Fig. S3 The phylogenetic tree compared to a matrix with the presence and absence of core and accessory genes (visualized using roary_plots.py script).

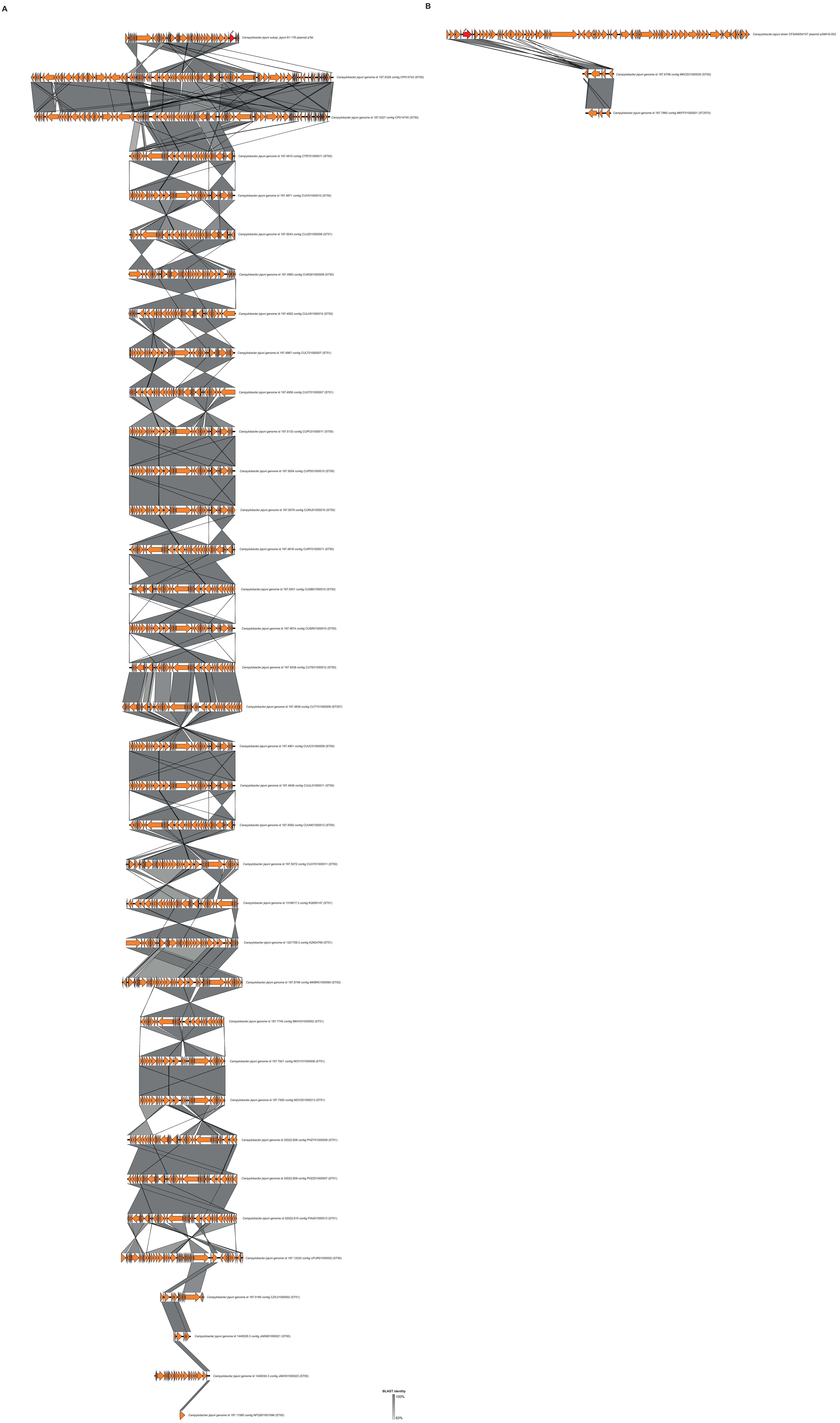

Fig. S4 Similarity of *C. jejuni* 81-176 plasmid pTet (A) and *C. jejuni* CFSAN054107 plasmid pGMI16-002 (B) with *C. jejuni* strains under the study carrying *tetO* on plasmid contigs.

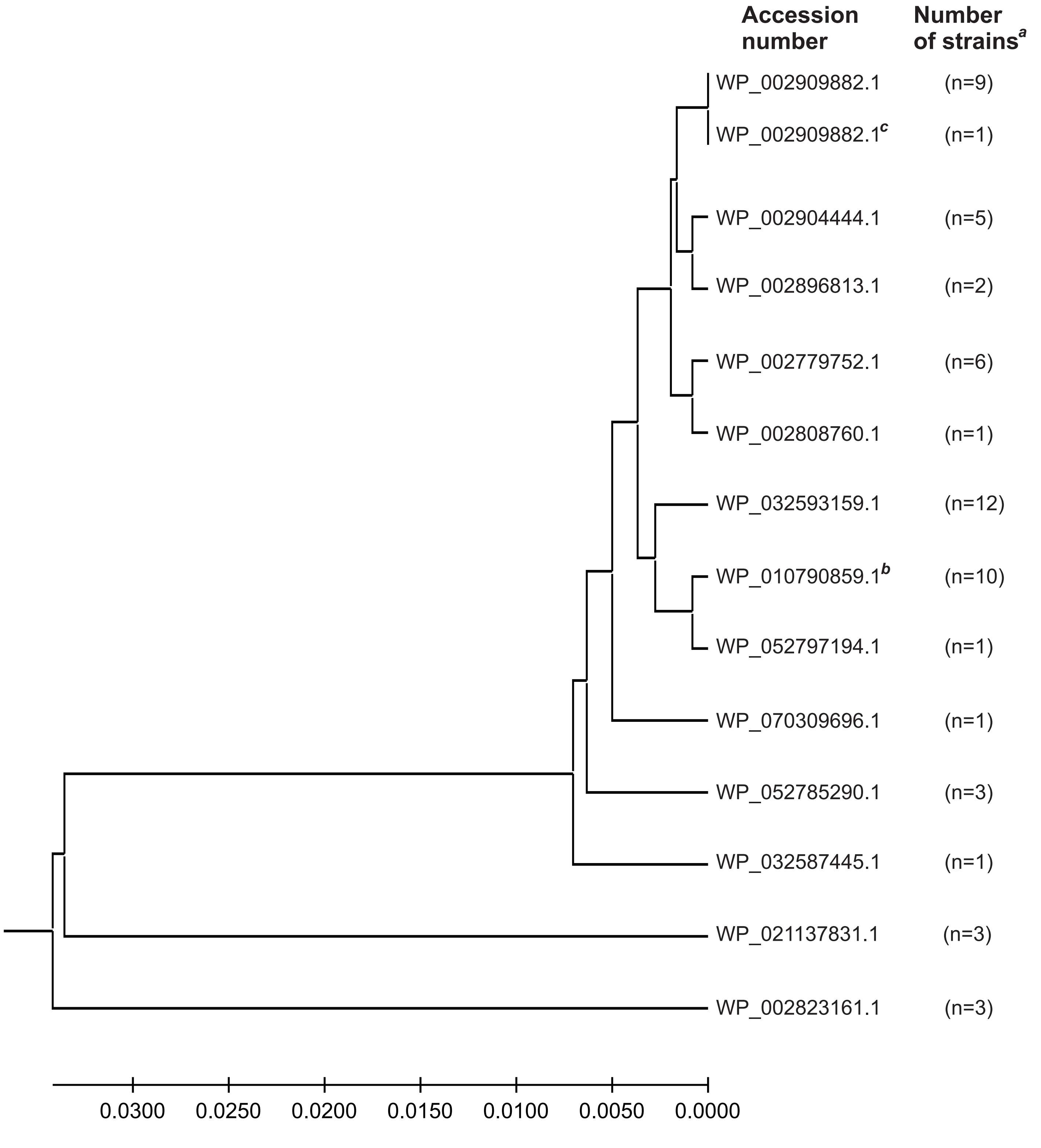

[a] one incomplete TetO sequence from *C. jejuni* strain 1440034.3 (ST50) was omitted

[b] TetO variant present in *C. jejuni* KF045

[c] TetO variant of WP_002909882.1 with additional alanine at position 534

Fig. S5 UPGMA cluster tree illustrating identity of amino acid sequences of TetO variants detected in *C. jejuni* strains.The evolutionary distances were computed using the Poisson correction method, and are in the units of the number of amino acid substitutions per site. The tree was built in MEGA7 software.

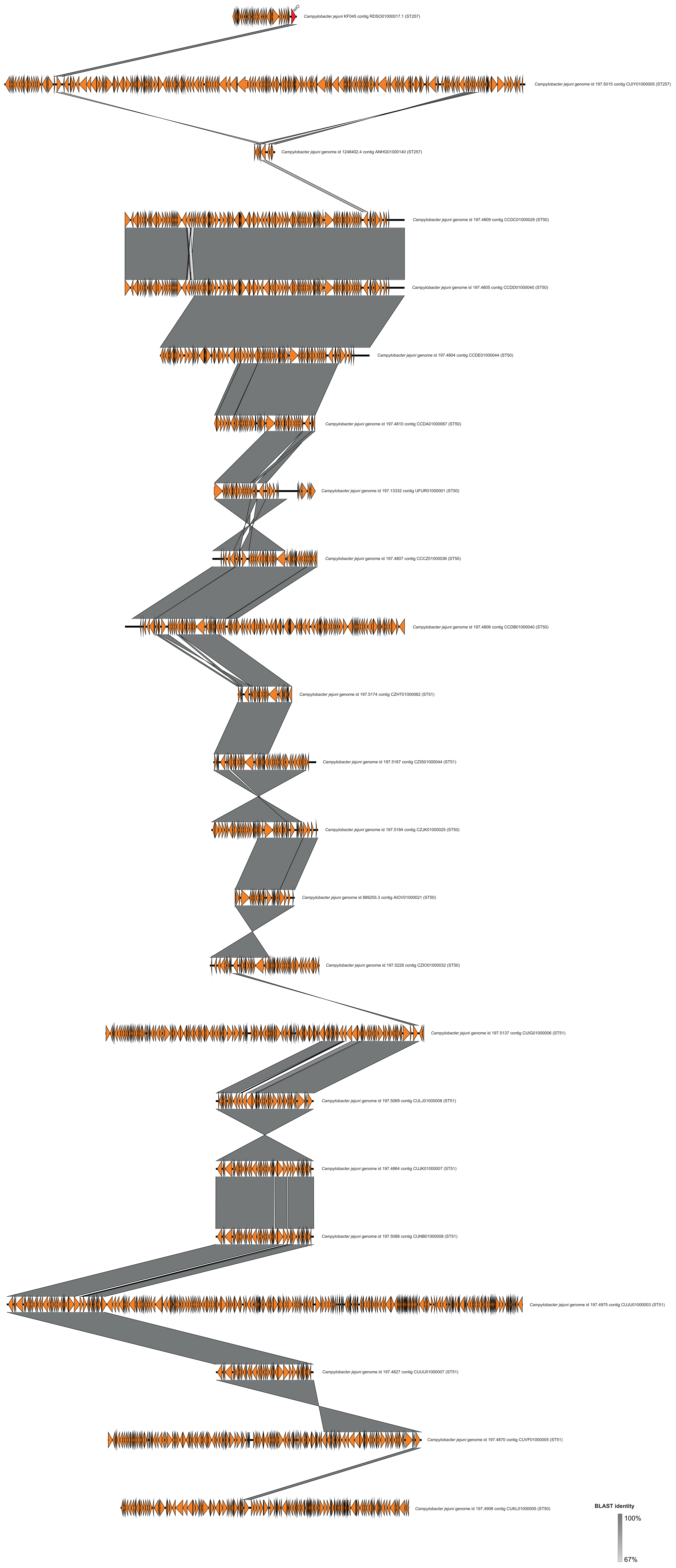

Fig. S6 Chromosomal locations of the *tetO* gene in *C. jejuni* genomes under the study.

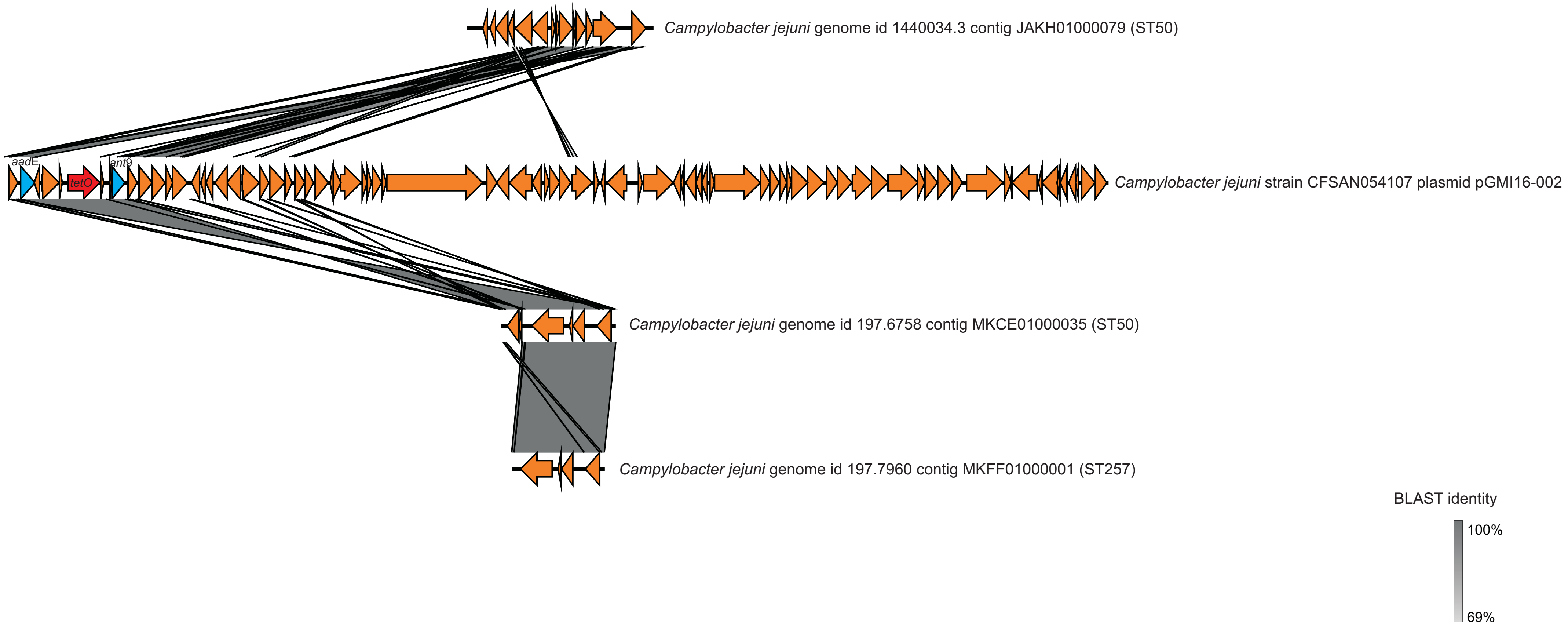

Fig. S7 Similarity of *C. jejuni* CFSAN054107 plasmid pGMI16-002 with *C. jejuni* genomes under the study carrying aminoglycoside resistance genes.
